# Anionic Effects on Lithium‐Ion Transport in Highly Concentrated Lithium Salt/Propylene Carbonate Solutions

**DOI:** 10.1002/cphc.70409

**Published:** 2026-05-15

**Authors:** Ryoichi Tatara, Kousuke Takeshita, Jiyoung Ock, Shuhei Miyazaki, Yosuke Ugata, Seiji Tsuzuki, Kaoru Dokko

**Affiliations:** ^1^ Department of Chemistry and Life Science Yokohama National University Yokohama Japan; ^2^ Advanced Chemical Energy Research Center (ACERC) Institute of Advanced Sciences Yokohama National University Yokohama Japan

**Keywords:** highly concentrated electrolyte, Lewis base, lithium‐ion transport, propylene carbonate, transference number

## Abstract

Highly concentrated electrolytes (HCEs) exhibit unique ion‐transport properties that fundamentally differ from those of conventional electrolytes; however, the role of anion species in governing Li^+^ transport remains unknown. Herein, Li^+^‐transport properties in lithium salt/propylene carbonate (LiX/PC) mixtures were systematically investigated by varying the basicity of the Lewis base anion: PF_6_^−^, N(SO_2_F)_2_^−^, N(SO_2_CF_3_)_2_^−^, ClO_4_^−^, BF_4_^−^, and SO_3_CF_3_^−^ (TfO^−^). Ionic conductivity, viscosity, self‐diffusion coefficients, and Li^+^ transference numbers under anion‐blocking conditions were evaluated and correlated with molecular‐scale structures obtained from molecular dynamics simulations. Weak Lewis‐base anions exhibited high ionic conductivity and coupled Li^+^‐solvent diffusion at high salt concentrations. Conversely, strong Lewis‐base anions promoted ion‐pair and aggregate formation, resulting in structural diffusion of Li^+^ and high transference numbers. Notably, Li^+^ transference numbers increased with anion Lewis basicity and concentration, attaining 0.83 for LiTfO/PC = 1/2.5, while conductivity decreased, revealing an intrinsic tradeoff between these transport descriptors. Therefore, anion Lewis basicity critically governs ion association, correlated motion, and Li^+^‐transport mechanisms in HCEs.

## Graphical abstract

This study systematically investigated the effects of anion species on Li^+^‐transport properties in highly concentrated lithium salt/propylene carbonate (LiX/PC) solutions (X = PF_6_, FSA, TFSA, ClO_4_, BF_4_, and TfO). Anion Lewis basicity controlled ion association and transport mechanisms, and practical high‐rate electrolytes require a balanced optimization of ionic conductivity and transference number.

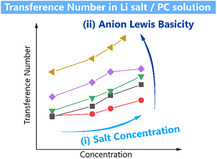

## 1 Introduction

As a key component of lithium‐ion batteries, electrolyte solutions govern ion transport between electrodes, determine interfacial reaction kinetics, and strongly influence power capability and durability [1, 2, 3]. In practical lithium‐ion batteries, nonaqueous electrolyte solutions typically comprise lithium salts dissolved in carbonate ester solvents at concentrations of ~1 mol dm^−3^ [4, 5, 6]. This widely adopted concentration optimally balances the increased number of ionic charge carriers with increasing salt concentration and a simultaneous increase in viscosity that reduces ionic mobility, which are two competing factors. Consequently, the ionic conductivity of conventional carbonate‐based electrolytes exhibits a maximum at approximately 1 mol dm^−3^, rendering this concentration range favorable for practical applications.

Lithium salts are generally more expensive than organic solvents, and increasing their concentration increases viscosity, lowers ionic conductivity, and decreases wettability. Thus, using large amounts of salt remains inefficient and limited for conventional lithium‐ion battery electrolytes. Recent studies have demonstrated that electrolyte solutions with salt concentrations of > 1 mol dm^−3^, referred to as highly concentrated electrolytes (HCEs), exhibit unique physicochemical and electrochemical properties [5, 6, 7, 8, 9, 10, 11]. In HCEs, the number of solvent molecules becomes insufficient to fully solvate Li^+^ ions, and anions directly participate in the Li^+^ coordination environment [12, 13]. This structural reorganization results in the formation of contact ion pairs (CIPs) and ionic aggregates (AGGs), yielding unique solvation structures that improve the electrochemical stability. Moreover, reductive stability is enhanced by the preferential reduction of anions involved in CIPs and AGGs, forming anion‐derived interphases that function as stable solid electrolyte interphases [14]. Simultaneously, the reduced population of free solvent molecules suppresses oxidative decomposition, thereby widening the electrochemical stability window compared with conventional electrolytes [15].

HCEs also exhibit fundamentally different ion‐transport properties. For example, in conventional electrolyte solutions, Li^+^ ions are solvated by multiple solvent molecules, forming bulky solvated cations whose hydrodynamic radii are typically larger than those of counter anions [4]. Consequently, Li^+^ ion mobility is lower than that of anions, and the Li^+^ transference number generally remains below 0.5. Conversely, in certain HCE systems, Li^+^ ions migrate through ligand‐exchange or hopping mechanisms within dynamically rearranging coordination networks comprising solvent molecules and anions [16]. Such transport behavior is typically observed when using solvents, such as sulfolane, capable of bidentate coordination and bridging multiple Li^+^ ions, or anions possessing relatively high Lewis basicity that readily form CIPs and ionic AGGs [6]. Under these conditions, Li^+^ ions diffuse faster than counter anions, resulting in unusually high Li^+^ transference numbers compared with those of conventional electrolytes. In electrolytes with high transference numbers, the concentration gradients are suppressed, thereby reducing concentration overpotential and enabling improved rate capability. Conversely, in those with low transference numbers, rapid charging or discharging induces concentration gradients across the electrolyte, resulting in severe concentration polarization. Therefore, an increased Li^+^ transference number is critically important for battery operation under high current densities [17]. However, the role of anion species in controlling ion transport in carbonate‐based HCEs has not yet been fully elucidated.

Herein, the effects of anion species on Li^+^‐transport properties in highly concentrated lithium salt/propylene carbonate (LiX/PC) solutions were systematically investigated. We previously reported the solvation structures and reaction kinetics evaluated with model electrodes using the same LiX/PC electrolyte systems [18]. Building on these earlier studies, we examined a series of lithium salts containing anions with distinctly different Lewis basicities: PF_6_^−^, bis(fluorosulfonyl)amide (N(SO_2_F)_2_^−^; FSA^−^), bis(trifluoromethanesulfonyl)amide (N(SO_2_CF_3_)_2_^−^; TFSA^−^), ClO_4_^−^, BF_4_^−^, and trifluoromethanesulfonate (SO_3_CF_3_^−^; TfO^−^). We aimed to clarify their influence on viscosity, ionic conductivity, self‐diffusion coefficients, and Li^+^ transference numbers. Molecular dynamics (MD) simulations of the LiX/PC mixtures were performed to investigate the Li^+^‐ion solvation structures. Our comprehensive approach provides fundamental insights into anion‐controlled ion transport in HCEs.

## 2 Experimental Section

### 2.1 Materials

Lithium bis(trifluoromethanesulfonyl)amide (LiTFSA) was purchased from Solvay Japan and dried overnight at 120°C. Battery‐grade PC, lithium bis(fluorosulfonyl)amide (LiFSA), LiPF_6_, LiBF_4_, and lithium trifluoromethanesulfonate (LiTfO) were purchased from Kishida Chemical, while battery‐grade LiClO_4_ was obtained from Sigma‐Aldrich. Electrolytes were prepared by mixing the lithium salts and PC in an argon‐filled glove box (VAC, [H_2_O] ~0.5 ppm).

### 2.2 Computational Methods

All *ab initio* molecular orbital calculations were performed using the Gaussian 16 program [19]. The basis set superposition error [20] was corrected for all intermolecular interaction energy calculations using the counterpoise method [21]. The Psi4 program [22] was used for the symmetry‐adapted perturbation theory calculations. Each energy term was calculated using the Symmetry‐Adapted Perturbation Theory (SAPT) based on Density Functional Theory (DFT) [23, 24]. MD simulations [25] were conducted using the MPDynPFF program [26, 27] with a polarizable force field [27]. Force field parameters were refined based on the calculated interaction energies and SAPT calculations. Details of the polarizable force field are presented in Table S1 and Figure S1 in Supporting Information, while those of the mathematical form of the polarizable force field are provided in the literature [27]. The force field parameters were refined to reproduce the interaction energies of Li^+^‐PC and Li^+^‐anion pairs obtained from the *ab initio* calculations, as shown in Figures S2 and S3. The MD simulations were performed for the following mixtures: LiPF_6_/PC, LiFSA/PC, LiTFSA/PC, LiClO_4_/PC, LiBF_4_/PC, and LiTfO/PC in the NPT ensemble (constant number of particles (N), pressure (P), and temperature (T)). All C–H bonds were constrained using the SHAKE/RATTLE algorithm [28]. The reversible reference system propagation algorithm was applied for the multitime step integration of the motion of atoms: 2 fs for bonded and short‐range interactions and 8 fs for long‐range Ewald interactions [29, 30]. Periodic boundary conditions were employed. The nonbonded forces were truncated at 12 Å, and the Coulomb interactions were computed using the Ewald summation method [25]. The Ewald alpha parameter was 0.238 Å^−1^. The Ewald summation tolerance was approximately 5 × 10^−6^. Constant temperature and pressure (0.1 MPa) conditions were maintained using a Nose–Hoover chain thermostat [31] and Andersen barostat, respectively [32]. The time constants of the thermostat and barostat were 0.5 and 2.0 ps, respectively. To minimize possible artifacts in the initial configuration, the system was pre‐equilibrated from initial structures under low‐density conditions for 1 ns at 403.15 K and 1 ns at 353.15 K before performing equilibration for 4 ns at 303.15 K. The density of the mixtures and radial distribution functions (RDFs) were evaluated from the 4 ns MD trajectories after the equilibration run at 303.15 K. The self‐diffusion coefficients of the ions and PC were calculated from the mean square displacement values obtained from 16 ns MD trajectories after the equilibration run at 303.15 K. The number of molecules used in each simulation was as follows: for the LiX(X = anion)/PC = 1/2.5 electrolyte: 76 LiPF_6_ and 190 PC; 72 LiFSA and 180 PC; 62 LiTFSA and 155 PC; 78 LiClO_4_ and 195 PC; 78 LiBF_4_ and 195 PC; and 74 LiTfO and 185 PC; for the LiX/PC = 1/8 electrolyte: 27 LiPF_6_ and 216 PC; 27 LiFSA and 216 PC; 25 LiTFSA and 200 PC; 28 LiClO_4_ and 224 PC; 28 LiBF_4_ and 224 PC; and 27 LiTfO and 216 PC.

### 2.3 Measurements

The viscosity and density of the electrolytes were measured using a Stabinger viscometer (SVM3001, Anton Paar), while the ionic conductivities of the electrolyte solutions were determined using an electrochemical station (VMP3, Biologic) and electrochemical impedance spectroscopy. The analysis was conducted in the frequency range of 500 kHz to 1 Hz with an amplitude of 10 mV root‐mean‐square. A cell containing two platinized platinum electrodes (CG‐511B, TOA Electronics) was utilized to measure the conductivity, and a KCl aqueous solution (0.01 mol dm^−3^) at 25°C was employed to determine the cell constant. Pulsed‐field gradient nuclear magnetic resonance (PFG‐NMR) spectra were obtained using a JEOL ECX‐400 NMR spectrometer, with a 9.4‐T narrow‐bore superconducting magnet equipped with a pulsed‐field gradient probe and current amplifier. To exclude the effects of convection, the electrolyte solution was injected to a height of approximately 5 mm into the NMR tube (BMS‐005J Shigemi). The diffusivities of different components of the solution, including the solvent, anions, and Li^+^ ions, were evaluated at 30°C. The detailed experimental procedure for obtaining the PFG‐NMR spectra has been previously reported [33].

To evaluate the Li^+^ transference number (*t*_Li+_^abc^) in the electrolyte solution under anion‐blocking conditions, a 2032‐type coin cell was used with a symmetric configuration of [Li | electrolyte/separator | Li]. The cell assembly was conducted in an argon‐filled glove box using a lithium–metal foil (thickness: 0.2 mm), purchased from Honjo Metal Co., cut to a diameter of 16 mm. A glass‐fiber filter (thickness: 200 µm; GA‐55, Advantec), cut into 17 mm pieces, was used as a separator. Electrochemical measurements were performed using an electrochemical station (VMP3, Biologic), and the temperature of the cell was controlled using a thermostat chamber (SU‐241). The chronoamperogram was recorded by applying a constant voltage (Δ*V* = 5 mV) to the Li/Li symmetric cell. The same cell was used for alternating current (AC) impedance measurements, with an amplitude of 10 mV, to determine the bulk electrolyte resistance (*R*_b_) and interfacial resistance at the lithium–metal electrodes. The AC impedance measurements were conducted before and after the chronoamperometry experiments. The *t*_Li+_^abc^ value was determined according to Equation (1) [34]:

(1) $${t_{\mathrm{Li}+}}^{\mathrm{abc}}=\frac{I_{\mathrm{ss}}\;(\text{Δ}V-I_{\Omega }\;R_{i,0})}{I_{\Omega }\;\left(\text{Δ}V-I_{\mathrm{ss}}\;R_{i,\mathrm{ss}}\right)}$$

where *I*_ss_ represents the steady‐state current observed in the chronoamperogram, *R*_i, 0_ and *R*_i, ss_ are the initial and steady‐state interfacial resistances at the lithium–metal electrode, respectively, and *I*_Ω_ is the Ohmic current, which was calculated from *R*_b_ and the potential drop across the electrolyte at the steady state: *I*_Ω_ = Δ*V*/(*R*_b_ + *R*_i, 0_).

## 3 Results and Discussion

### 3.1 Li^+^ Transport in the LiX/PC Mixtures

**FIGURE 1:**
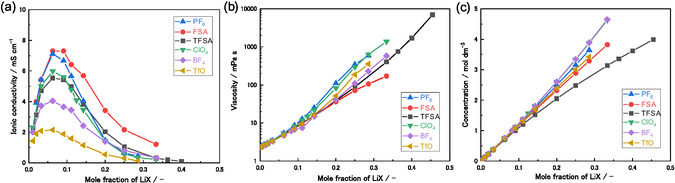
(a) Ionic conductivity, (b) viscosity, and (c) Li^+^ concentration as a function of LiX mole fraction for the LiX/PC solutions measured at 30°C. The corresponding plots presented as a function of molar concentration are shown in Figure S4, and all numerical data are summarized in Table S2.

**FIGURE 2:**
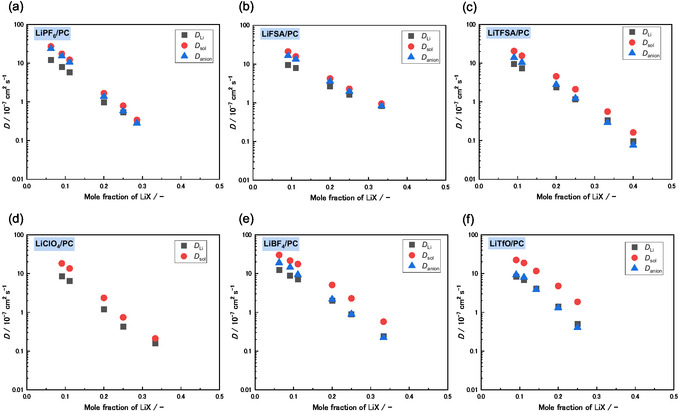
Self‐diffusion coefficients of Li^+^ (*D*_Li_), PC (*D*_PC_), and the anion (*D*_anion_) as a function of the LiX mole fraction in (a) LiPF_6_/PC, (b) LiFSA/PC, (c) LiTFSA/PC, (d) LiClO_4_/PC, (e) LiBF_4_/PC, and (f) LiTfO/PC solutions at 30°C. All numerical data are summarized in Table S2. *D*_anion_ values are not available for the LiClO_4_/PC electrolyte because PFG‐NMR measurements of chloride nuclei are not feasible. The self‐diffusion coefficients of *D*_Li_, *D*_PC_, and *D*_anion_ plotted individually are shown in Figure S5.

The conductivity, viscosity, and molar concentration of the LiX/PC electrolytes as a function of the LiX mole fraction are presented in Figure 1a–c. For each salt, the electrolyte solutions were prepared and evaluated up to their respective solubility limits in PC. The ionic conductivity of the LiX/PC electrolytes exhibited a clear maximum at a mole fraction of 0.063 (LiX/PC = 1/15), corresponding to ~0.7 mol dm^−3^ (Figure 1a and Table S2). This result was consistent with those for electrolyte systems reported in the literature [35, 36, 37, 38, 39]. Increasing the LiX mole fraction up to ~0.063 increased the number of charge carriers in the electrolyte, thereby enhancing the ionic conductivity. Conversely, the viscosity of each electrolyte monotonically increased with increasing salt concentration (Figure 1b), which reduced ion mobility and resulted in a decrease in the self‐diffusion coefficients of all species (Figure 2). At higher salt contents (*X*_LiX_ > 0.1), the decrease in ion mobility dominated over the increase in charge carrier concentration, resulting in a volcano‐shaped dependence of ionic conductivity on salt concentration (Figure 1a).

Among the lithium salts examined, the ionic conductivity trended as follows: FSA^−^ ≃ PF_6_^−^ > TFSA^−^ ≃ ClO_4_^−^ > BF_4_^−^ > TfO^−^. This phenomenon was attributed to the combined effect of anion size and Lewis basicity. Based on the donor number, computational studies, and spectroscopic evidence previously reported, the Lewis basicity of these anions is as follows: PF_6_^−^ ≃ FSA^−^ ≃ TFSA^−^ < ClO_4_^−^ < BF_4_^−^ < TfO^−^ [18]. The PF_6_^−^ and FSA^−^ ions are relatively small, which was intrinsically favorable for fast ion transport. Moreover, they exhibited weak Lewis basicity that suppressed ion‐pair formation, resulting in high ionic conductivity. TFSA^−^ also exhibited weak Lewis basicity; however, its larger ionic size resulted in lower conductivity compared with PF_6_^−^ and FSA^−^. Conversely, the ClO_4_^−^ and BF_4_^−^ ions are relatively small; however, they exhibited stronger Lewis basicity, which promoted ion‐pair and AGG formation and, consequently, reduced ionic conductivity. TfO^−^ exhibited the strongest Lewis basicity among the studied anions and has a relatively large ionic size, resulting in the lowest ionic conductivity.

These anion‐dependent trends were similar to those for viscosity (Figure 1b) and molar concentration (Figure 1c). For small anions, such as ClO_4_^−^ and BF_4_^−^, the volumetric molar concentration became higher at the same mole fraction owing to their smaller ionic volumes (Figure 1c). However, the viscosity trends were more complex and can be understood as the combination of three distinct effects. First, small anions, such as BF_4_^−^, ClO_4_^−^, and PF_6_^−^, have higher volumetric molar concentrations at the same salt mole fraction, which intrinsically increase viscosity owing to the increased number density of ionic species. Second, large anions, such as TFSA^−^, tend to increase the viscosity by enlarging the effective hydrodynamic radius of the charge carriers. Third, anions with high Lewis basicity, such as TfO^−^, readily form ion pairs or ionic AGGs, thereby increasing the hydrodynamic radius and enhancing viscosity. Consequently, FSA^−^, which combines a moderately small ionic size with weak Lewis basicity, exhibited the lowest viscosity among the investigated electrolytes.

**FIGURE 3:**
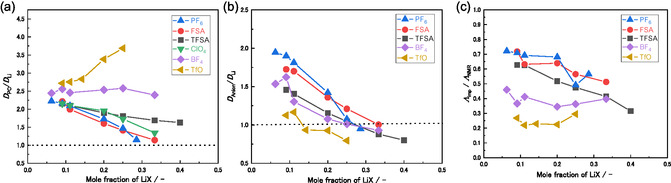
Diffusivity ratios (a) *D*_PC_/*D*_Li_, (b) *D*_anion_/*D*_Li_, and (c) ratio of molar ionic conductivity determined from AC impedance compared to that determined using PFG‐NMR (*Λ*_imp_/*Λ*_NMR_) as a function of the LiX mole fraction in LiX/PC solutions at 30°C. All numerical data are summarized in Table S2. The *D*_anion_/*D*_Li_ and *Λ*_imp_/*Λ*_NMR_ values are not available for the LiClO_4_/PC electrolyte because PFG‐NMR measurements of chloride nuclei are not feasible.

The mechanism of ion transport was further investigated by measuring the self‐diffusion coefficients of Li^+^ (*D*_Li_), the anions (*D*_anion_), and PC (*D*_PC_) using PFG‐NMR. Figure 2 presents a summary of the absolute values of diffusion coefficients as a function of LiX concentration. For all electrolytes, *D*_Li_, *D*_anion_, and *D*_PC_ decreased from 10^−6^ to 10^−8^ cm^2^ s^−1^ with increasing salt concentration, reflecting the increase in viscosity at higher concentrations (Figure 1b). The diffusion coefficients organized by individual species (*D*_Li_, *D*_anion_, and *D*_PC_) are shown in Figure S5. At low salt concentrations, the diffusion coefficients were nearly independent of the anion and exhibited comparable values among *D*_Li_, *D*_anion_, and *D*_PC_. Conversely, at high salt concentrations, the LiFSA/PC mixtures showed consistently higher *D*_Li_, *D*_anion_, and *D*_PC_ values compared to those of the other electrolytes. This observation is consistent with the viscosity trends shown in Figure 1b. Figure 3a,b show the ratios of diffusion coefficients *D*_PC_/*D*_Li_ and *D*_anion_/*D*_Li_, respectively. With increasing salt concentration, *D*_PC_/*D*_Li_ generally decreased for all electrolytes, except for LiTfO/PC and LiBF_4_/PC, which contained the strongest Lewis basic anions among the salts examined.

The solvation of Li^+^ ions with PC molecules resulted in the formation of solvated complexes, [Li(PC)_*n*_]^+^, where the coordination number of Li^+^ in nonaqueous electrolytes is generally 4–5 [40]. In the electrolytes with LiX mole fractions below ~0.2 (LiX/PC > 1/4), both coordinated and non‐coordinated PC molecules coexisted. Although these PC molecules could not be distinguished using PFG‐NMR owing to the rapid exchange of solvent molecules in the first solvation shell of Li^+^, the residence time of PC was shorter than the NMR timescale. Therefore, the measured *D*_PC_ value represented an average diffusion coefficient of coordinated and free PC molecules. In a continuous medium, diffusion coefficients are inversely proportional to the hydrodynamic (Stokes) radius. Accordingly, solvent molecules coordinated to Li^+^ ions exhibit lower diffusivity than free solvent molecules. This behavior was clearly observed, as shown in Figure 2, where *D*_Li_ is smaller than *D*_PC_ in the dilute region. With increasing salt concentration, *D*_PC_/*D*_Li_ generally decreased for LiPF_6_/PC, LiFSA/PC, LiTFSA/PC, and LiClO_4_/PC, reflecting a reduction in the fraction of free PC molecules [41, 42, 43]. Notably, in the highly concentrated LiFSA/PC electrolyte at *X*_LiFSA_ = 0.33, *D*_PC_/*D*_Li_ approached unity, indicating that the Li^+^ ions diffused at similar rates to those of PC molecules. For electrolytes containing weak Lewis‐base anions, increasing the salt concentration reduced the number of PC molecules that were not coordinated to Li^+^ ions. Consequently, Li^+^ ions and coordinating solvent molecules increasingly diffused in a coupled manner, resulting in comparable diffusion coefficients for Li^+^ and PC at high salt concentrations. The solvation environment around Li^+^ ions was governed by competitive coordination between the solvent molecules and anions. As the Lewis basicity of the anion increased, the anions tended to directly coordinate to Li^+^ ions, thereby displacing solvent molecules from the first solvation shell. Consequently, the excluded PC molecules independently diffused from the Li^+^ ions. This effect was pronounced in electrolytes containing strong Lewis‐base anions, such as BF_4_^−^ and TfO^−^, where the number of free PC molecules was high even at high salt concentrations, resulting in increased *D*_PC_/*D*_Li_ values (Figure 3a). These observations were consistent with our previous Raman spectroscopic studies on the same LiX/PC systems [18], which demonstrated enhanced anion coordination to Li^+^ and reduced solvent participation in the first solvation shell as the anion Lewis basicity increased.

Figure 3b presents the *D*_anion_/*D*_Li_ ratios for the LiX/PC electrolytes. For those containing weak Lewis‐base anions, such as TFSA^−^, the hydrodynamic radius of the anion was smaller than that of the solvated Li^+^ ions, resulting in a faster anion diffusion compared with Li^+^. At low salt concentrations, *D*_anion_/*D*_Li_ was higher than unity for all electrolytes, indicating that the anions diffused faster than the Li^+^ ions. As the salt concentration increased, the populations of CIPs and AGGs increased [18], and a larger fraction of anions participated in Li^+^ coordination. Because Li^+^ ions dynamically exchange coordinating anions, PFG‐NMR detects only averaged diffusion coefficients of the free and coordinated anions. Consequently, increasing the salt concentration resulted in a decrease in *D*_anion_/*D*_Li_. In the dilute region, *D*_anion_/*D*_Li_ tended to increase with decreasing Lewis basicity of the anions. Among the weak Lewis‐base anions, *D*_anion_/*D*_Li_ increased as follows: TFSA^−^ < FSA^−^ < PF_6_^−^. This occurred because the smaller ionic sizes of PF_6_^−^ and FSA^−^ resulted in faster diffusion compared to the larger ions. For strongly Lewis basic anions, such as TfO^−^, *D*_anion_/*D*_Li_ was low owing to the formation of anion‐bridged AGG structures, in which a single anion coordinated multiple Li^+^ ions. At high concentrations (*X*_Li_ > 0.33, LiX/PC ≧ 1/2), *D*_anion_/*D*_Li_ was lower than unity for all electrolytes; thus, the Li^+^ ions diffused faster than the anions. This inversion of relative diffusivities has been reported for sulfone‐based HCEs. Such dynamic ligand exchange within the solvation network facilitates Li^+^ hopping conduction in HCEs [16, 44, 45, 46]. This anomalous diffusion behavior likely originated from the formation of aggregated structures, such as [Li_*x*_(PC)_*y*_(anion)_*z*_]^x−z^, in which Li^+^–anion–Li^+^ bridging occurs, and Li^+^ diffusion is promoted through ligand exchange. Particularly, for the LiTfO/PC electrolyte, *D*_anion_/*D*_Li_ was below unity at *X*_LiTfO_ = 0.143 (LiTfO/PC ≧ 1/6); thus, the formation of AGGs and resulting Li^+^ hopping conduction occurred at lower salt concentrations owing to the highest Lewis basicity of TfO^−^. This interpretation was further supported by our previous Raman spectroscopic analysis, which directly evidenced the formation of AGGs in LiTfO/PC electrolytes at concentrations higher than *X*_LiTfO_ = 0.125–0.25 (LiTfO/PC ≧ 1/8–1/4) [18].

As shown in Figure 3c, the ion‐transport properties were evaluated by comparing the molar ionic conductivity determined using AC impedance spectroscopy (*Λ*_imp_) with that estimated from the self‐diffusion coefficients obtained using PFG‐NMR (*Λ*_NMR_). *Λ*_NMR_ is calculated from the Nernst–Einstein relation, *Λ*_NMR_ = *F* ^2^(*D*_Li_ + *D*_anion_)/*RT*, where *F* is the Faraday constant, *R* is the gas constant, and *T* is the absolute temperature. *Λ*_NMR_ is a theoretical value calculated under the assumption of ideal ionic dissociation, in which all ionic species independently contribute to charge transport and no correlated motion between ions occurs. Often referred to as ionicity, the ratio *Λ*_imp_/*Λ*_NMR_ reflects the correlated motion of ions in the electrolyte and is equivalent to the inverse of the Haven ratio [47, 48, 49]. As shown in Figure 3c, *Λ*_imp_/*Λ*_NMR_ increased as the Lewis basicity of the anion decreased, which is consistent with the ionic conductivity order observed in Figure 1a and the anion‐dependent ionicity trends reported in the literature [17]. When the Lewis basicity of the anion was low, such as for FSA^−^ and TFSA^−^, ion‐pair formation was suppressed, and correlated ionic motion (i.e., the concerted diffusion of cations and anions in the same direction) was less pronounced. Consequently, *Λ*_imp_/*Λ*_NMR_ attained relatively high values. Conversely, for strong Lewis‐base anions that readily formed ion pairs, such as BF_4_^−^ and TfO^−^, correlated motion became significant, thereby reducing the *Λ*_imp_/*Λ*_NMR_ value. With increasing salt concentration, *Λ*_imp_/*Λ*_NMR_ gradually decreased for electrolytes containing weakly Lewis basic anions, such as TFSA^−^, FSA^−^, and PF_6_^−^. Thus, ion‐pair formation and the associated correlated motion became increasingly important. Conversely, for the strong Lewis‐base BF_4_^−^ and TfO^−^ anions, *Λ*_imp_/*Λ*_NMR_ showed little dependence on salt concentration. The formation of AGGs at high salt concentrations in electrolytes containing strong Lewis‐base anions [18] promoted Li^+^ diffusion *via* ligand‐exchange mechanisms. Such transport processes increased the contribution of anticorrelated ionic motion, thereby mitigating the reduction in *Λ*_imp_/*Λ*_NMR_ despite the high degree of ion association.

### 3.2 Li^+^ Coordination Estimated Using MD Simulations

**FIGURE 4:**
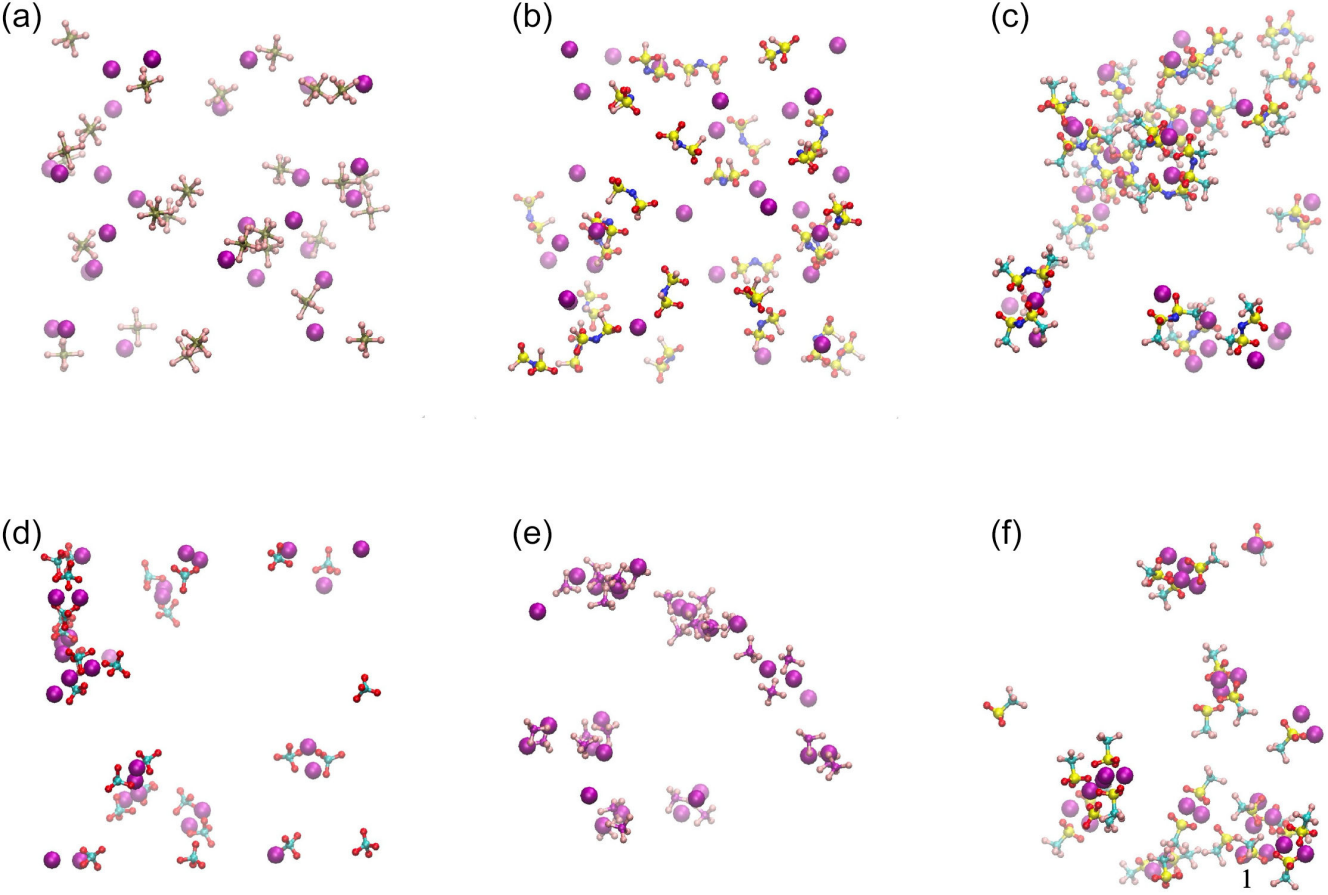
Snapshots of the dilute solutions of LiX (LiX/PC = 1/8). Note: PC molecules are omitted. Carbon, oxygen, nitrogen, fluorine, sulfur, phosphorus, chlorine, boron, and lithium atoms are shown in light blue, red, blue, pink, yellow, brown, blue, and purple, respectively. Li^+^ ions are represented using a space‐filling model, whereas other atoms are shown using a ball‐and‐stick model: (a) X = PF_6_; (b) X = FSA; (c) X = TFSA; (d) X = ClO_4_; (e) X = BF_4_; and (f) X = TfO.

MD simulations were performed to investigate the liquid structures of electrolytes with LiX/PC ratios of 1/2.5 and 1/8, where X = PF_6_, FSA, TFSA, ClO_4_, BF_4_, and TfO. Figure 4 and Figure S6–S11 show their simulated structures at 303.15 K. For LiPF_6_/PC and LiFSA/PC, no aggregation of Li^+^ ions and anions was observed in either the dilute (LiX/PC = 1/8) or concentrated (LiX/PC = 1/2.5) solutions. Conversely, for LiTFSA/PC, LiClO_4_/PC, LiBF_4_/PC, and LiTfO/PC, multiple Li^+^ ions and anions formed clusters, and aggregation was observed in both the dilute and concentrated solutions. Although no significant phase separation was observed in LiPF_6_/PC and LiFSA/PC, microphase separations of LiX and PC were observed in other systems.

**FIGURE 5:**
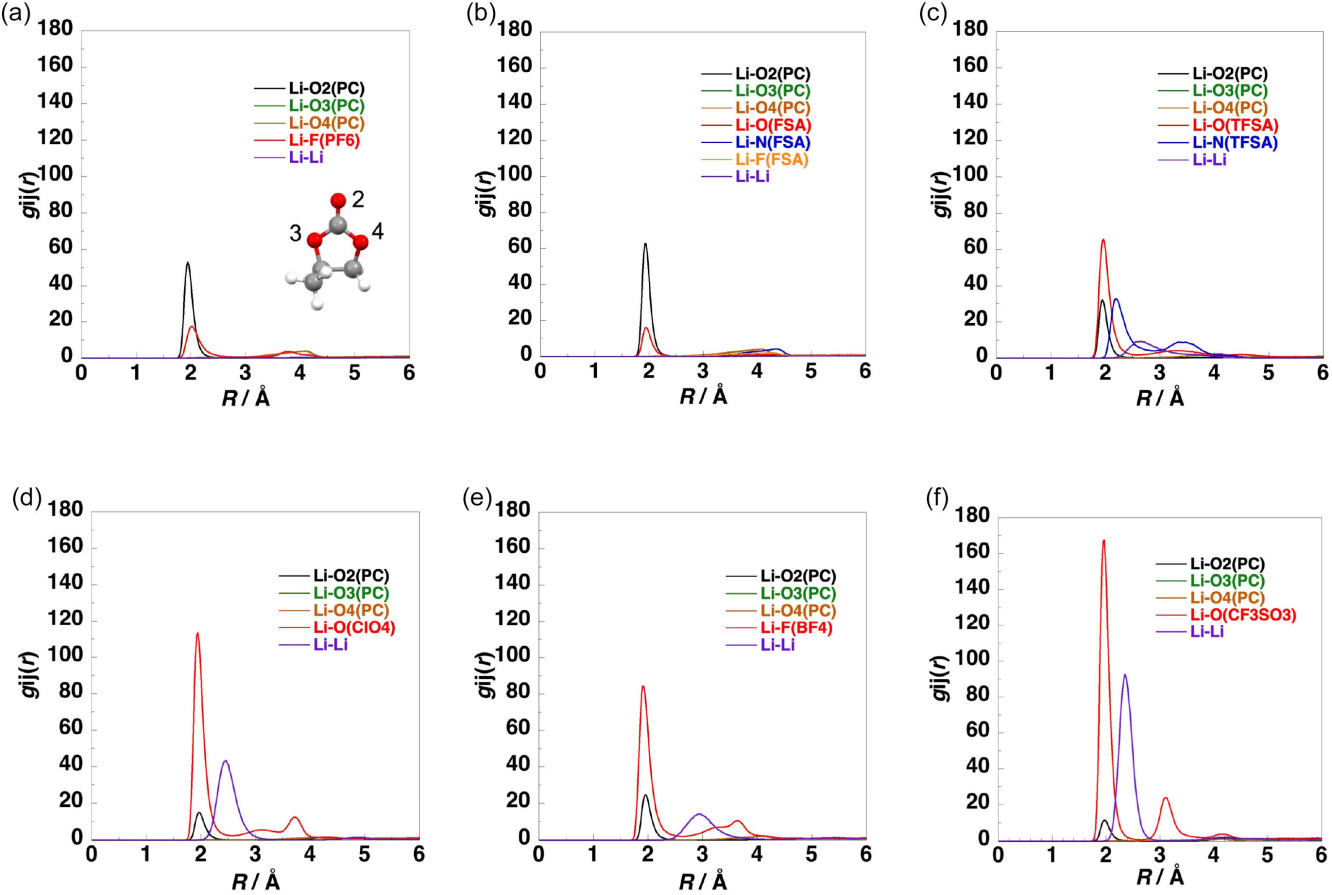
Site–site intermolecular RDFs for dilute solutions of LiX (LiX/PC = 1/8): (a) X = PF_6_; (b) X = FSA; (c) X = TFSA; (d) X = ClO_4_; (e) X = BF_4_; and (f) X = TfO.

The site–site intermolecular RDFs obtained from the MD simulations at 303.15 K are shown in Figure 5 and Figure S12. For all electrolytes, the RDFs between Li^+^ ions and the carbonyl oxygen atoms of PC (O2) exhibited sharp peaks at ~2 Å, indicating strong coordination between them. However, the RDFs between Li^+^ ions and other oxygen atoms of PC (O3 and O4) did not show peaks at ~2 Å, indicating that these oxygen atoms did not coordinate with Li^+^ ions.

The RDFs between Li^+^ ions and the oxygen atoms of the FSA, TFSA, ClO_4_, and TfO anions; the fluorine atoms of the PF_6_ and BF_4_ anions; and the nitrogen atoms of the TFSA anions showed peaks at ~2 Å, indicating that these anionic atoms coordinated with the Li^+^ ions. Conversely, the RDFs between Li^+^ ions and the nitrogen and fluorine atoms of the FSA anions did not indicate coordination. In both the dilute and concentrated solutions of LiPF_6_/PC and LiFSA/PC, the carbonyl oxygen peak of PC was higher than those corresponding to the anionic oxygen, fluorine, and nitrogen atoms. Conversely, for the electrolytes containing other lithium salts, the anionic oxygen and fluorine peaks were higher than the carbonyl oxygen peak of PC.

**FIGURE 6:**
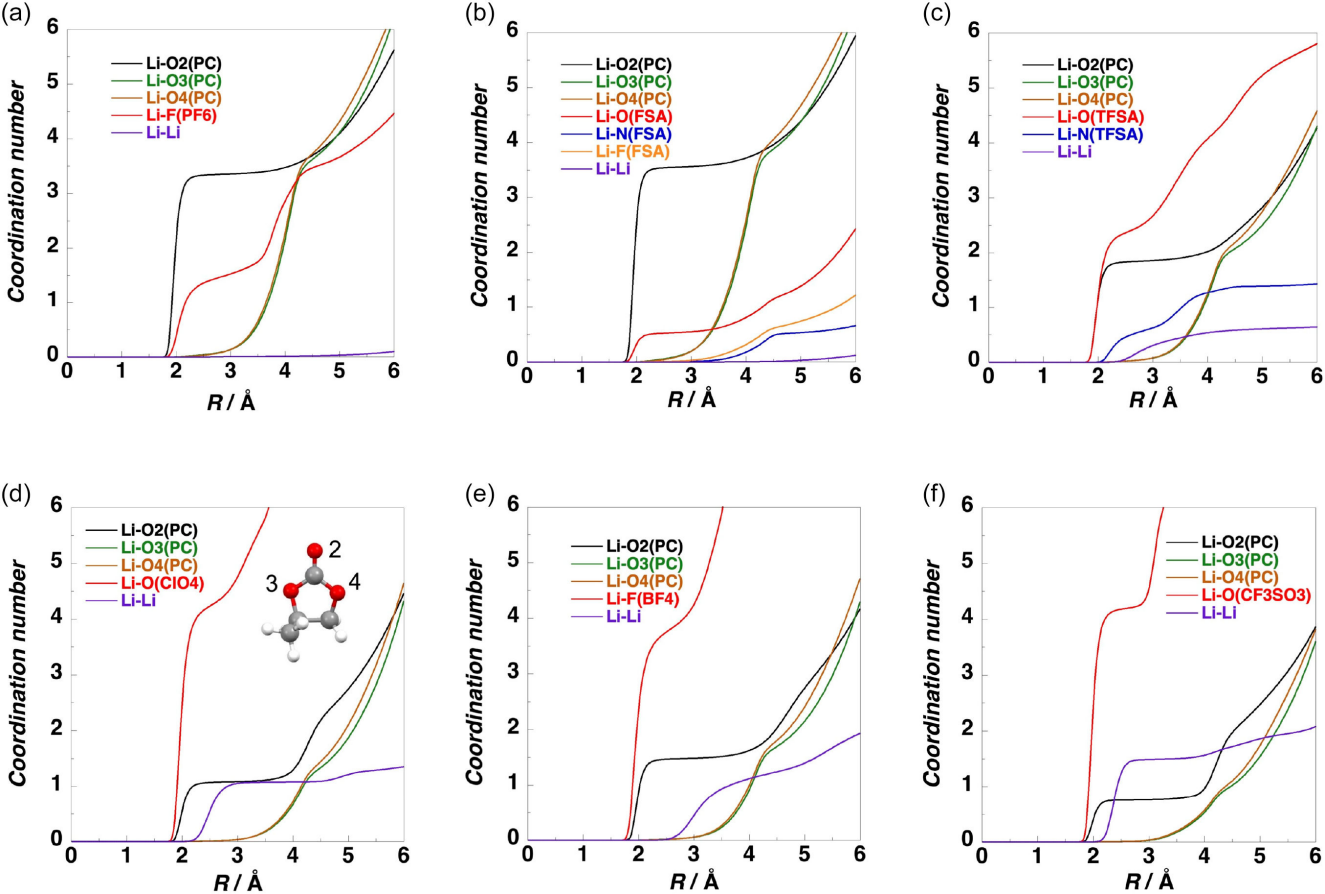
Cumulative coordination numbers around Li^+^ ions for dilute solutions of LiX (LiX/PC = 1/8): (a) X = PF_6_; (b) X = FSA; (c) X = TFSA; (d) X = ClO_4_; (e) X = BF_4_; and (f) X = TfO.

Figure 6 and Figure S13 show the cumulative coordination numbers of the oxygen atoms of PC and the oxygen, fluorine, and nitrogen atoms of anions around Li^+^ ions in the electrolytes. In both the dilute and concentrated solutions of LiFSA/PC and LiTFSA/PC, approximately four oxygen atoms were coordinated with each Li^+^ ion. Conversely, in those of the other lithium salts, approximately five atoms, comprising the carbonyl oxygen atoms of PC and the oxygen or fluorine atoms of the anions, coordinated with each Li^+^ ion.

In dilute solutions, the cumulative coordination number of the carbonyl oxygen atoms of PC increased compared with that in concentrated solutions. In the dilute LiPF_6_/PC and LiFSA/PC solutions, the cumulative numbers of the carbonyl oxygen atoms of PC were larger than those of the oxygen and fluorine atoms of the anions, indicating that Li^+^ ions preferentially coordinate to PC rather than to the anions. In LiFSA/PC, the coordination numbers of the carbonyl oxygen atoms of PC and the oxygen atoms of FSA at 2.5 Å were 3.5 and 0.5, respectively. This result indicates that anion coordination is minimal, suggesting that solvent‐separated ion pairs (SSIPs) are predominantly formed, while the formation of CIPs and AGGs is negligible. In LiPF_6_/PC, the corresponding coordination numbers of the carbonyl oxygen atoms of PC and the fluorine atoms of PF_6_^−^ at 2.5 Å were 3.3 and 1.4, respectively. The larger coordination number of PF_6_^−^ compared with FSA^−^ indicates enhanced formation of CIPs in LiPF_6_/PC relative to LiFSA/PC. In other LiX solutions containing strongly Lewis basic anions, the coordination numbers of the anions exceeded those of the carbonyl oxygen atoms of PC. For example, in LiTFSA/PC, LiClO_4_/PC, LiBF_4_/PC, and LiTfO/PC, the coordination numbers of the carbonyl oxygen atoms of the PC were 1.8, 1.1, 1.7, and 0.8, respectively, whereas those of the anions were 2.4, 4.2, 3.4, and 4.2, respectively. These results indicate that Li^+^ ions preferentially coordinate to the anions rather than to PC, leading to predominant formation of CIPs and AGGs in these systems.

In the concentrated solutions, a similar dependence of the cumulative coordination numbers on the Lewis basicity of the anions was observed. The cumulative coordination number of oxygen, nitrogen, and fluorine atoms of the anions increased compared with that in dilute solutions. The cumulative coordination numbers of the carbonyl oxygen atoms of PC at 2.5 Å in LiPF_6_/PC, LiFSA/PC, LiTFSA/PC, LiClO_4_/PC, LiBF_4_/PC, and LiTfO/PC were 2.1, 2.2, 1.0, 0.9, 1.5, and 0.6, respectively, while the corresponding coordination numbers of the oxygen and fluorine atoms of the anions were 3.0, 1.9, 3.1, 4.4, 3.7, and 4.4, respectively. In LiFSA/PC, the coordination number of the carbonyl oxygen atoms of PC (2.2) was comparable to that of the oxygen atoms of FSA (1.9), whereas in other systems, the coordination numbers of the anions exceeded those of the carbonyl oxygen atoms of PC. These results indicate that in the concentrated solutions, Li^+^ ions preferentially coordinate to the anions, leading to predominant formation of CIPs and AGGs.

The RDFs of the LiPF_6_/PC and LiFSA/PC solutions did not exhibit Li–Li peaks near 2.5 Å, while in those of LiX containing anions with a high Lewis basicity, distinct Li–Li peaks were observed near 2.5 Å in both the dilute and concentrated solutions. Moreover, the cumulative Li–Li coordination number increased from approximately 2.5 Å. Thus, Li^+^ ions and anions formed AGGs in the LiX solutions with highly Lewis basic anions. Notably, in the LiTfO/PC solution, the cumulative Li–Li coordination number was large, suggesting the formation of large Li^+^–anion clusters.

The calculated densities of the dilute solutions (Table S3) agreed with the experimental values, with errors of < 3%. The errors in the calculated densities of the concentrated solutions of LiTFSA/PC, LiBF_4_/PC, and LiTfO/PC were also < 3%, while those of the concentrated solutions of LiFSA/PC, LiPF_6_/PC, and LiClO_4_/PC were higher (3%–6%).

In both the dilute and concentrated solutions, the experimental self‐diffusion coefficient of PC was higher than that of the Li^+^ ions and anions. However, the magnitudes of the self‐diffusion coefficients of Li^+^ ions and anions were not significantly different. The calculated self‐diffusion coefficients were similar to the experimental trend. Except for the LiClO_4_/PC solution, the calculated self‐diffusion coefficients of PC and ions in dilute solutions were 20%–70% lower than the experimental values. Even in the concentrated LiFSA/PC and LiTfO/PC solutions, the calculated self‐diffusion coefficients of PC and the ions were 10%–70% lower than the experimental values. Conversely, in concentrated solutions of the other LiX species, the calculated self‐diffusion coefficients of PC and the ions were higher than the experimental values. Because the calculated densities of these concentrated solutions were lower than the experimental values, the self‐diffusion coefficients were likely overestimated.

### 3.3 Li^+^ Transference Number

**FIGURE 7:**
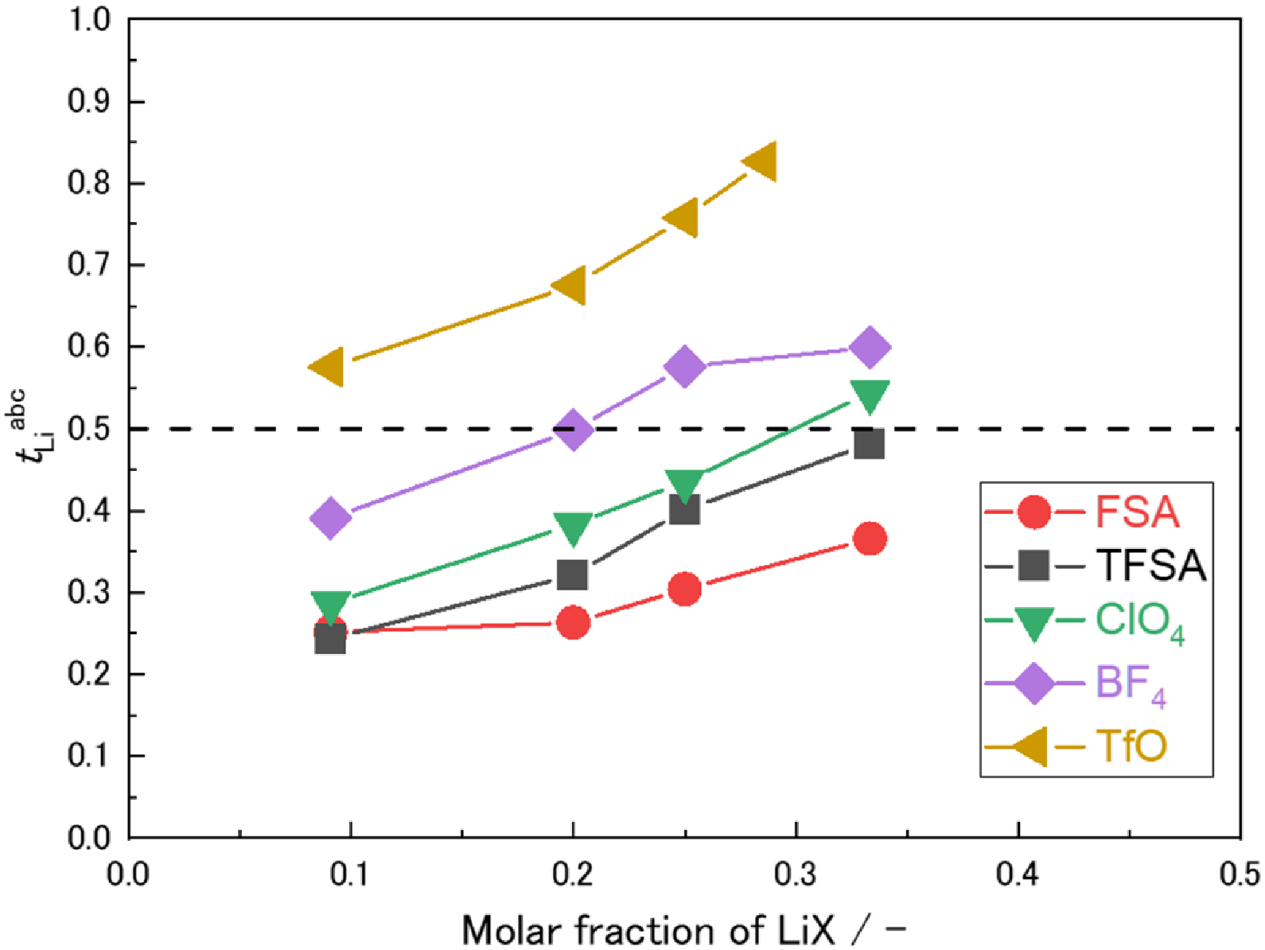
Li^+^ transference number under anion‐blocking conditions, *t*_Li+_^abc^, as a function of the LiX mole fraction in LiX/PC solutions at 30°C. The Nyquist plots and chronoamperograms used to determine *t*_Li+_^abc^ are shown in Figures S14–S18. Measurements at LiTfO/PC = 1/2 (*X*_Li_ = 0.333) were not performed because the electrolyte was in a solid state.

Figure 7 shows the Li^+^ transference number under anion‐blocking conditions, *t*_Li+_^abc^, as a function of the LiX mole fraction in LiX/PC solutions using a Li/Li symmetric cell at 30°C. LiPF_6_/PC was excluded from this evaluation owing to its poor stability against Li metal in the highly concentrated regime, which is consistent with a previous report on concentrated LiPF_6_/sulfone electrolytes [50]. For other electrolytes, both the chronoamperograms and the Nyquist plots before and after polarization remained stable, as shown in Figures S14–S18. Therefore, the analysis of *t*_Li+_^abc^ is not significantly affected, as Equation (1) explicitly accounts for and removes the contribution of interfacial resistance. Nevertheless, the relationship between concentrated electrolyte composition and the stability of the Li metal interface should be further investigated in future work.

Unlike the diffusivity ratio *D*_anion_/*D*_Li_ obtained from PFG‐NMR (Figure 3b), which does not account for dynamic correlation effects between ions, *t*_Li+_^abc^ represents the effective contribution of Li^+^ ions to the steady‐state ionic current under anion‐blocking conditions and inherently reflects correlated ionic motion. Therefore, *t*_Li+_^abc^ provides complementary information on Li^+^ transport that cannot be captured using self‐diffusion measurements. For LiX/PC = 1/10 (*X*_Li_ = 0.091), *t*_Li+_^abc^ systematically increased with increasing Lewis basicity of the anion as follows: FSA^−^ = TFSA^−^ < ClO_4_^−^ < BF_4_^−^ < TfO^−^. As the Lewis basicity of the anion increased, the population of neutral ion pairs that did not contribute to charge transport also increased, resulting in a decrease in ionic conductivity (Figure 1a). Conversely, the order of *t*_Li+_^abc^ was opposite that of the ionic conductivity trends, highlighting a clear tradeoff between these two transport parameters.

As the salt concentration increased, *t*_Li+_^abc^ showed an increasing trend for all anions. Even at high concentrations, *t*_Li+_^abc^ remained higher for electrolytes containing stronger Lewis basic anions (TfO^−^ > BF_4_^−^ > ClO_4_^−^ > TFSA^−^ > FSA^−^). In particular, LiTfO/PC exhibited exceptionally high *t*_Li+_^abc^ values, attaining 0.83 for LiTfO/PC = 1/2.5 (*X*_Li_ = 0.286). Conversely, electrolytes containing weakly Lewis basic anions, such as FSA and TFSA, showed *t*_Li+_^abc^ values below 0.5 even at LiX/PC = 1/2 (*X*_Li_ = 0.33). These observations agreed with previous studies on glyme‐ and sulfolane‐based electrolytes [51]. Weak Lewis‐base anions, such as FSA^−^ and TFSA^−^, promoted anticorrelated motion between the Li^+^ ions and anions, reducing the *t*_Li+_^abc^ value under anion‐blocking conditions. In such electrolytes, Li^+^ ions predominantly migrated as PC‐coordinated species, and the resulting large effective mass and hydrodynamic size of the solvated Li^+^ complexes enhanced anticorrelated motion, owing to the constraint of momentum conservation. Conversely, strong Lewis‐base anions, such as BF_4_^−^ and TfO^−^, tended to form ionic AGGs with Li^+^ ions in the HCEs. Notably, Li^+^ ions could readily hop between neighboring coordination sites. These AGGs induced pronounced correlated motion between Li^+^–Li^+^ and Li^+^–anion pairs, resulting in high *t*_Li+_^abc^ values under anion‐blocking conditions. Because *t*_Li+_^abc^ is closely linked to the formation of CIPs and AGGs, a fundamental tradeoff between ionic conductivity and *t*_Li+_^abc^ was observed. Particularly, electrolytes exhibiting higher *t*_Li+_^abc^ values tended to show lower ionic conductivity, indicating that the enhancement of *t*_Li+_^abc^ was achieved at the expense of overall ionic conductivity within the current design framework.

## 4 Conclusion

This study comprehensively investigated Li^+^‐transport properties in LiX/PC mixtures. The ionic conductivity of the electrolyte increased with increasing LiX mole fraction up to approximately 0.063 (LiX/PC = 1/15, ~0.7 mol dm^−3^) owing to an increased number of charge carriers in the solution. However, the viscosity of the electrolyte also monotonically increased with increasing salt concentration, exhibiting a volcano‐shaped behavior in the conductivity–concentration curve. Among the various lithium salts, LiFSA/PC exhibited the highest ionic conductivity, followed by those with the PF_6_^−^, TFSA^−^, ClO_4_^−^, BF_4_^−^, and TfO^−^ anions. The low ionic conductivity of the BF_4_^−^‐ and TfO^−^‐based electrolytes was attributed to their high Lewis basicity and the aggregation of lithium salts in the electrolyte solution. With increasing salt concentration, *D*_PC_/*D*_Li_ decreased for weak Lewis‐base anions, indicating that coupled Li^+^ and PC molecule diffusion as free solvent molecules were depleted. Conversely, strongly Lewis basic anions promoted anion coordination and increased the fraction of free PC molecules, resulting in higher *D*_PC_/*D*_Li_ values. *D*_anion_/*D*_Li_ decreased with increasing electrolyte concentration and became lower than unity at high concentrations, indicating that the diffusion of Li^+^ was faster than that of the anions. In particular, LiTfO/PC exhibited AGG formation even at relatively low salt fractions. The *Λ*_imp_/*Λ*_NMR_ ratio confirmed that weakly Lewis basic anions suppressed ion pairing and correlated motion in dilute solutions, while aggregation and ligand‐exchange transport were important at high concentrations. The MD simulations revealed that the solvation structures of the Li^+^ ions in PC solutions strongly depended on the anion Lewis basicity. In both the dilute and concentrated solutions, Li^+^ ions and the anions formed AGGs in LiX solutions containing highly Lewis basic anions. Conversely, the LiX solutions did not form AGGs when the basicity of the anions was low. Finally, *t*_Li+_^abc^ increased with increasing anion Lewis basicity and salt concentration, attaining high values for LiTfO/PC (0.83 for LiTfO/PC = 1/2.5). Therefore, anion Lewis basicity controls ion association and transport mechanisms in carbonate‐based HCEs. Moreover, practical high‐rate electrolytes require a balanced optimization of ionic conductivity and transference number under the current design framework.

## Supporting Information

Additional supporting information can be found online in the Supporting Information section.

## Funding

This study was supported by Japan Society for the Promotion of Science (JP22H00340, JP23K17370, JP25H01967), Japan Science and Technology Agency (JPMJGX23S0, JPMJPR2374, JPMJAP2528).

## Conflicts of Interest

The authors declare no conflicts of interest.

## Supporting information

Supplementary Material

## Data Availability

The data that support the findings of this study are available from the corresponding author upon reasonable request.
