# Supplementary material for: Anionic Effects on Lithium‐Ion Transport in Highly Concentrated Lithium Salt/Propylene Carbonate Solutions
Source: Chemphyschem. 2026 May 15;27(9):e70409. doi: 10.1002/cphc.70409 (PMC13176805; doi:10.1002/cphc.70409)
Supplement: Supplementary file 1 — Supplementary Material [file CPHC-27-e70409-s001.pdf]

Supporting Information

Anionic Effects on Lithium-Ion Transport in Highly Concentrated Lithium Salt/Propylene Carbonate Solutions

Ryoichi Tatara,[1, 2] Kousuke Takeshita,[1] Jiyoung Ock,[1] Shuhei Miyazaki,[1]

*Yosuke Ugata,[1, 2] Seiji, Tsuzuki,[2] and Kaoru Dokko[1, 2]**

[1]Department of Chemistry and Life Science, Yokohama National University,79-5 Tokiwadai, Hodogaya-ku, Yokohama 240-8501, Japan

[2]Advanced Chemical Energy Research Center (ACERC), Institute of Advanced Sciences, Yokohama National University, Yokohama 240-8501, Japan

*To whom correspondence should be addressed.

Telephone/Fax: +81-45-339-3942. E-mail: dokko-kaoru-js@ynu.ac.jp

Table S1 Force field parameters used for the molecular dynamics (MD) simulations. The atom types are explained in **Figure S1**.

Nonbonding parameters

Atom	σ (Å)	ε (kcal mol^{-1})	α (a.u.)
CS	3.50	0.066	8.0
CT	3.50	0.066	8.0
CB	3.75	0.105	9.0
OB	2.96	0.21	8.0
OR	3.10	0.14	4.0
HC	2.50	0.03	1.0
NI	3.55	0.17	7.4
SO	3.55	0.25	16.0
OS	3.00	0.13	5.0
CF	3.50	0.066	8.0
FC	3.15	0.053	2.5
OST	3.0	0.13	5.0
FS	3.05	0.053	5.3
OSU	3.05	0.13	6.4
PF	3.742	0.20	20.0
FP1	3.2	0.061	4.5
FP2	3.2	0.061	4.5
FP3	3.2	0.061	4.5
BF	3.5814	0.095	10.0
FB	3.0	0.061	3.5
CLO	3.58	0.095	10.0
OCL	3.2	0.061	6.0

$E_{\text{nonbond}} = 4\varepsilon\,[(\sigma/r)^{12} - (\sigma/r)^{6}]$

Bond stretching parameters

Bond	k_s (kcal mol^{-1} Å^{-2})	r_0 (Å)
CS–CS	268.0	1.529
CS–CT	268.0	1.529
CS–HC	340.0	1.09
CT–HC	340.0	1.09
CS–OR	570.0	1.433
CB–OR	570.0	1.360
CB–OB	570.0	1.193
NI–SO	744.0	1.60
SO–OS	1274.0	1.45

SO–CF	471.0	1.835
CF–FC	884.0	1.34
SO–OST	1274.0	1.45
SO–FS	884.0	1.64
SO–OSU	1274.0	1.45
PF–FP1	190.0	1.628
PF–FP2	190.0	1.628
PF–FP3	190.0	1.628
BF–FB	290.0	1.412
CLO–OCL	290.0	1.459

$E_{str} = k_s (r - r_0)^2$

Angle bending parameters

Angle	k_θ (kcal mol^{-1} rad^{-2})	θ_0 (deg)
CS–CS–CT	58.35	112.7
CS–CS–HC	37.5	110.7
CT–CS–HC	37.5	110.7
CS–CT–HC	37.5	110.7
HC–CS–HC	33.0	107.8
HC–CT–HC	33.0	107.8
CS–CS–OR	80.0	109.0
CT–CS–OR	80.0	109.0
HC–CS–OR	35.0	109.5
CS–OR–CB	55.0	114.0
OR–CB–OR	81.0	108.0
OR–CB–OB	83.0	122.0
SO–NI–SO	80.0	121.0
NI–SO–OS	189.0	111.4
NI–SO–CF	195.0	100.2
OS–SO–OS	232.0	120.2
OS–SO–CF	208.0	102.6
SO–CF–FC	166.0	110.4
FC–CF–FC	187.0	108.6
NI–SO–OST	189.0	111.4
NI–SO–FS	189.0	103.5
OST–SO–OST	232.0	120.2
OST–SO–FS	189.0	104.5
CF–SO–OSU	208.0	102.6
OSU–SO–OSU	232.0	120.2

FP1–PF–FP2	80.0	90.0
FP1–PF–FP3	80.0	90.0
FP2–PF–FP3	80.0	90.0
FP1–PF–FP1	0.0	180.0
FP2–PF–FP2	0.0	180.0
FP3–PF–FP3	0.0	180.0
FB–BF–FB	50.0	109.5
OCL–CLO–OCL	50.0	109.5

$E_{bend} = k_\theta(\theta - \theta_0)^2$

Torsional parameters

Dihedral	V_1 (kcal mol^{-1})	V_2 (kcal mol^{-1})	V_3 (kcal mol^{-1})
CT–CS–CS–OR	1.743	0.157	0.279
OR–CS–CS–OR	4.0	4.0	0.4
CT–CS–CS–HC			0.366
OR–CS–CS–HC			0.366
HC–CS–CS–HC			0.318
CS–CS–CT–HC			0.366
OR–CS–CT–HC			0.366
HC–CS–CT–HC			0.318
CT–CS–OR–CB	−1.220	−0.126	0.422
CS–CS–OR–CB	−1.220	−0.126	0.422
HC–CS–OR–CB			0.198
OB–CB–OR–CS		−5.124	
OR–CB–OR–CS		−5.124	
CF–SO–NI–SO	7.833	1.500	−0.764
OS–SO–NI–SO			−0.004
FC–CF–SO–NI			0.3
FC–CF–SO–OS			0.171
FS–SO–NI–SO	−1.120	1.000	−0.359
OST–SO–NI–SO			−0.004
FC–CF–SO–OSU			0.171

$E_{torsion} = \Sigma\, V_n/2\ (1 + \cos(n\varphi))$

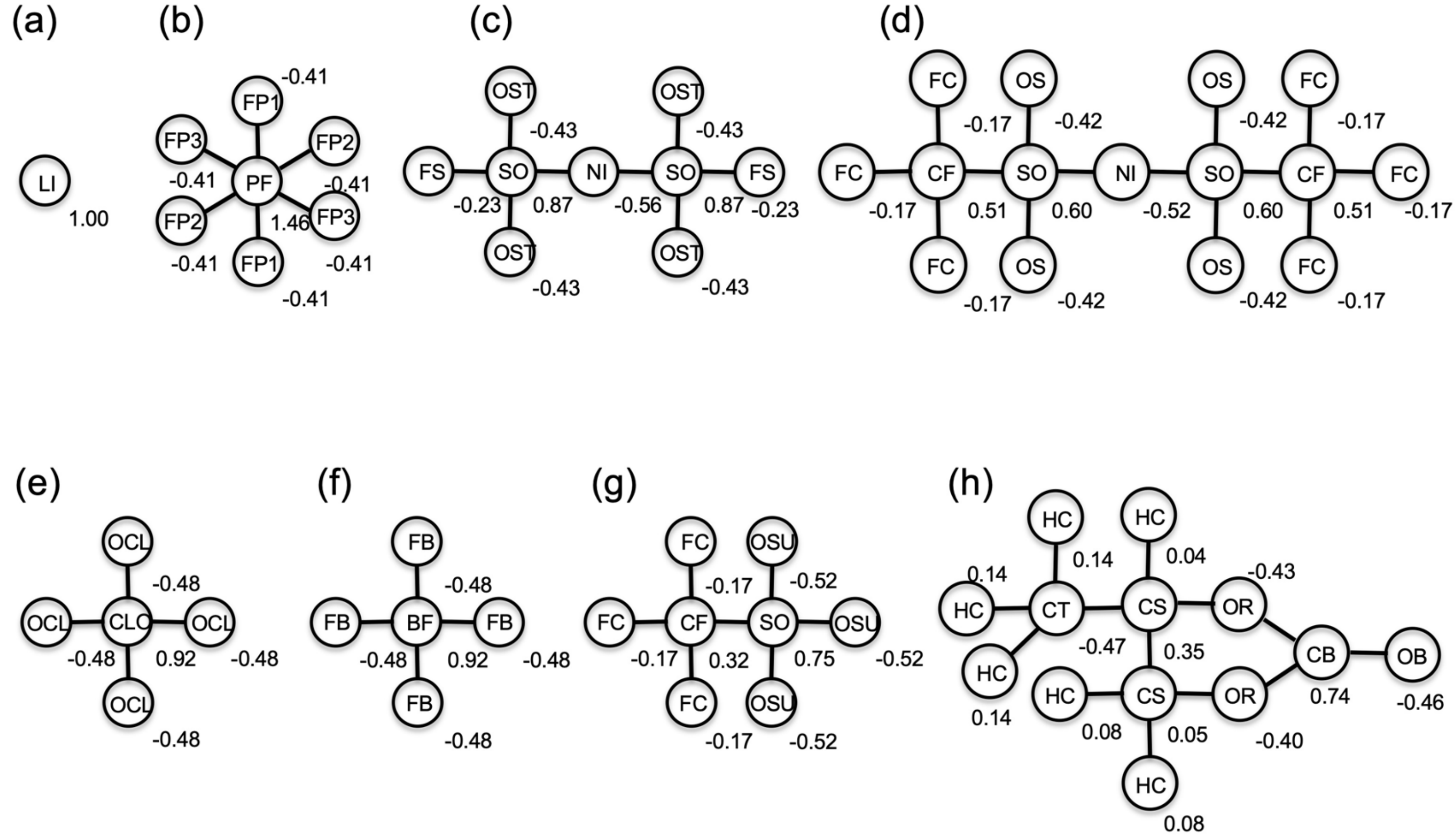

Figure S1 Atom types and charges used for the MD simulations: (a) Li^{+}; (b) $[PF_6]^{-}$; (c) $[FSA]^{-}$; (d) $[TFSA]^{-}$; (e) ClO_4^{-}; (f) BF_4^{-}; (g) $[TfO]^{-}$; and (h) propylene carbonate (PC).

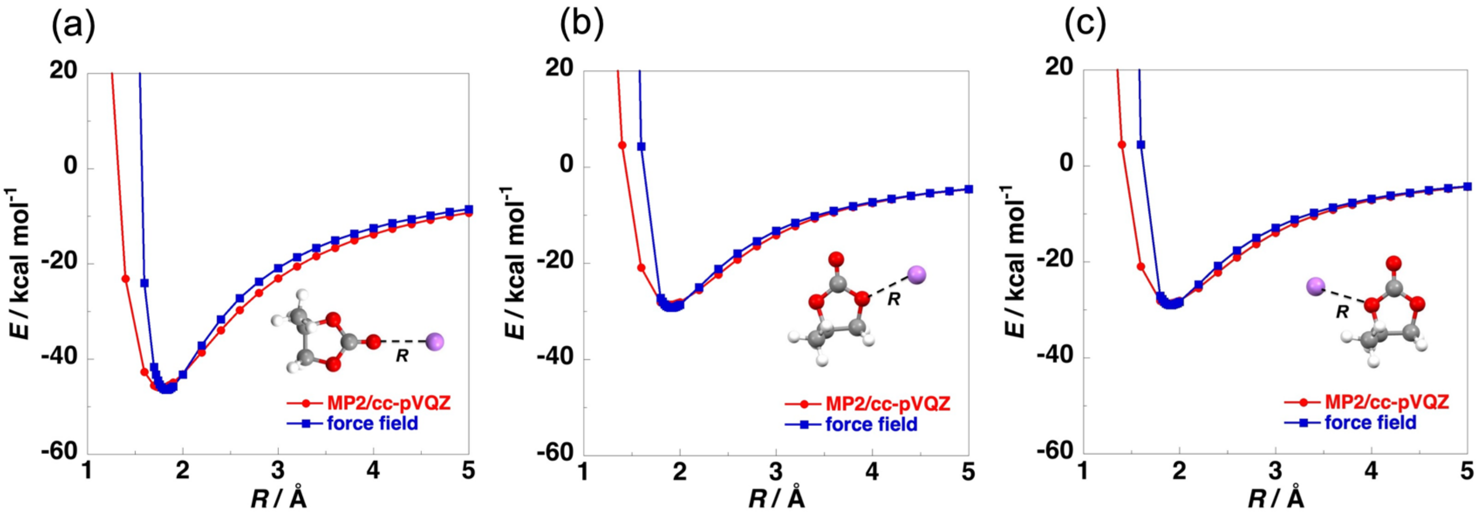

Figure S2 Comparison of the interaction energy potentials of Li^+ with PC obtained using MP2/cc-pVQZ level *ab initio* and force field calculations with changing Li···O distance (*R*): (a) Li^+ is located on the extension of the C–O bond; and (b), (c) Li^+ is located on the bisector of the C–O–C angle.

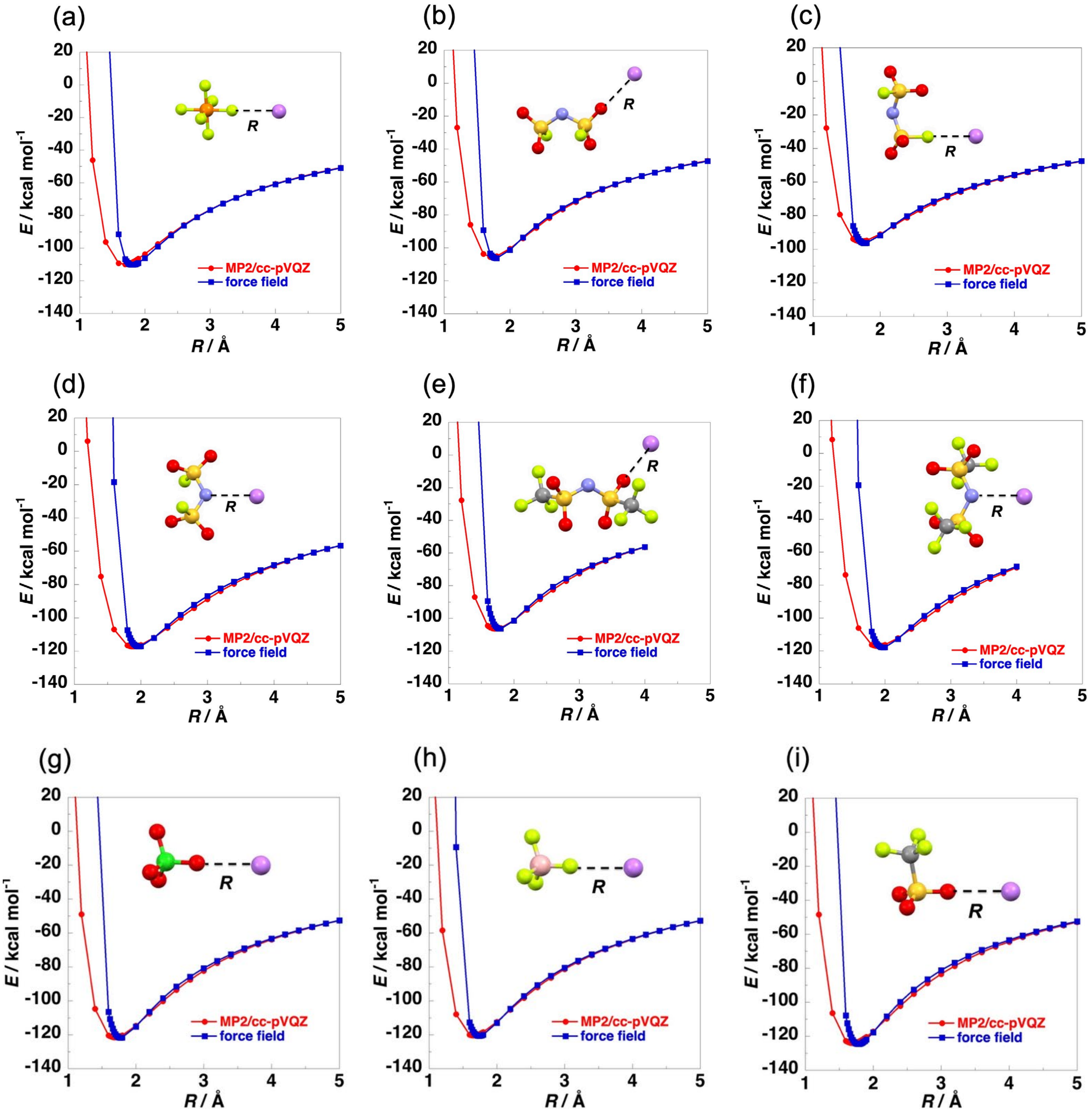

Figure S3 Comparison of the interaction energy potentials of Li^+ with anions obtained using MP2/cc-pVQZ level *ab initio* and force field calculations with changing Li···O (or F, N) distance (*R*): (a) Li^+ is located on the extension of the P–F bond of PF_6^-; (b)–(d) Li^+ is located on the extension of the S–O or S–F bond or on the bisector of the S–N–S angle of $[FSA]^-$; (e), (f) Li^+ is located on the extension of the S–O bond or on the bisector of the S–N–S angle of $[TFSA]^-$; (g) Li^+ is located on the extension of the Cl–O bond of ClO_4^-; (h) Li^+ is located on the extension of the B–F bond of BF_4^-; and (i) Li^+ is located on the extension of the S–O bond of $[OfT]^-$n.

Table S2 Physicochemical properties of the investigated electrolyte solutions at 30 ℃.

	Mole fraction	Viscosity	Density	Concentration	Conductivity	Λ_{imp}	D_{Li}	D_{sol}	D_{anion}*	Λ_{NMR}	$\Lambda_{imp}/\Lambda_{NMR}$	t_{Li}^{abc}
		mPa s	g cm^{-3}	mol dm^{-3}	mS cm^{-1}	S cm^2 mol^{-1}		10^{-7} cm^2/s		S・cm^2/mol		
$LiPF_6$/PC=1/2.5	0.286	584	1.483	3.643	0.44	0.12	0.30	0.34	0.28	0.21	0.57	
$LiPF_6$/PC=1/3	0.250	346	1.446	3.155	0.64	0.20	0.54	0.79	0.58	0.41	0.49	
$LiPF_6$/PC=1/4	0.200	111	1.395	2.491	1.47	0.59	0.97	1.67	1.38	0.87	0.68	
$LiPF_6$/PC=1/6	0.143	25	1.339	1.752	4.03	2.30						
$LiPF_6$/PC=1/8	0.111	13	1.306	1.348	5.65	4.19	5.83	12.21	10.57	6.06	0.69	
$LiPF_6$/PC=1/10	0.091	8.7	1.285	1.096	6.67	6.09	8.02	17.36	15.23	8.59	0.71	
$LiPF_6$/PC=1/15	0.063	5.4	1.256	0.746	7.11	9.53	12.14	26.97	23.61	13.20	0.72	
$LiPF_6$/PC=1/30	0.032	3.4	1.226	0.381	5.43	14.23						
$LiPF_6$/PC=1/50	0.020	3.0	1.213	0.231	3.90	16.91						
LiFSA/PC=1/2	0.333	171	1.495	3.821	1.205	0.32	0.83	0.95	0.83	0.62	0.51	0.366
LiFSA/PC=1/3	0.250	71	1.425	2.889	2.161	0.75	1.62	2.29	1.96	1.32	0.57	0.304
LiFSA/PC=1/4	0.200	36	1.382	2.321	3.416	1.47	2.64	4.23	3.59	2.30	0.64	0.263
LiFSA/PC=1/6	0.143	15	1.333	1.666	5.685	3.41						
LiFSA/PC=1/8	0.111	9.6	1.301	1.296	6.413	4.95	7.9	15.7	13.4	7.83	0.63	
LiFSA/PC=1/10	0.091	7.1	1.284	1.063	7.302	6.87	9.5	21.0	16.4	9.56	0.72	0.252
LiFSA/PC=1/15	0.063	4.9	1.257	0.731	7.299	9.98						
LiFSA/PC=1/30	0.032	3.4	1.226	0.377	5.417	14.36						
LiFSA/PC=1/50	0.020	2.9	1.214	0.229	3.988	17.38						
LiTFSA/PC=1/1.5	0.400	1707	1.592	3.617	0.10	0.03	0.10	0.16	0.08	0.06	0.42	
LiTFSA/PC=1/1.75	0.364	756	1.565	3.360	0.19	0.06						
LiTFSA/PC=1/2	0.333	402	1.540	3.135	0.30	0.10	0.33	0.56	0.29	0.23	0.41	0.482
LiTFSA/PC=1/3	0.250	89	1.472	2.481	1.05	0.42	1.17	2.12	1.23	0.89	0.48	0.401
LiTFSA/PC=1/4	0.200	39	1.425	2.049	2.02	0.99	2.40	4.55	2.77	1.91	0.52	0.322
LiTFSA/PC=1/6	0.143	16	1.368	1.521	3.82							
LiTFSA/PC=1/8	0.111	10	1.332	1.207	4.97	4.12	7.40	15.50	10.40	6.58	0.63	
1M LiTFSA/PC	0.091	7.5	1.310	1	5.42	5.42	9.52	20.51	13.89	8.65	0.63	0.243
LiTFSA/PC=1/15	0.063	5.0	1.275	0.701	5.54	7.90						
LiTFSA/PC=1/30	0.032	3.4	1.237	0.369	4.70							
LiTFSA/PC=1/50	0.020	2.9	1.221	0.226	3.12	13.79						
LiTFSA/PC=1/90	0.011	2.6	1.209	0.128	2.28							
$LiClO_4$/PC=1/2	0.333	1379	1.437	4.628	0.22	0.05	0.16	0.21				0.544
$LiClO_4$/PC=1/2.5	0.286	631	1.404	3.882	0.34	0.09						
$LiClO_4$/PC=1/3	0.250	294	1.377	3.336	0.58	0.17	0.43	0.74				0.436
$LiClO_4$/PC=1/4	0.200	83	1.340	2.604	1.43	0.55	1.21	2.36				0.383
$LiClO_4$/PC=1/6	0.143	22	1.300	1.808	3.46	1.91						
$LiClO_4$/PC=1/7	0.125	15	1.285	1.565	4.09	2.61						
$LiClO_4$/PC=1/8	0.111	12	1.275	1.381	4.79	3.46	6.45	13.54				
$LiClO_4$/PC=1/10	0.091	8.2	1.260	1.118	5.59	5.00	8.49	18.29				0.287
$LiClO_4$/PC=1/15	0.063	5.3	1.239	0.757	5.984	7.91						
$LiClO_4$/PC=1/30	0.032	3.4	1.218	0.384	5.01	13.05						
$LiClO_4$/PC=1/100	0.010	2.6	1.201	0.116	2.31	19.84						
$LiBF_4$/PC=1/2	0.333	581	1.385	4.651	0.32	0.07	0.24	0.57	0.22	0.17	0.40	0.599
$LiBF_4$/PC=1/3	0.250	110	1.338	3.344	0.80	0.24	0.89	2.29	0.90	0.66	0.36	0.576
$LiBF_4$/PC=1/4	0.200	41	1.309	2.607	1.38	0.53	2.00	5.08	2.16	1.54	0.35	0.499
$LiBF_4$/PC=1/6	0.143	15	1.277	1.809	2.42	1.34						
$LiBF_4$/PC=1/8	0.111	7.4	1.250	1.373	3.44	2.51	7.16	17.61	9.34	6.09	0.41	
$LiBF_4$/PC=1/10	0.091	6.8	1.246	1.118	3.65	3.27	8.93	21.58	14.50	8.65	0.38	0.391
$LiBF_4$/PC=1/15	0.063	4.7	1.232	0.758	4.04	5.33	12.37	30.26	19.00	11.59	0.46	
$LiBF_4$/PC=1/30	0.032	3.3	1.213	0.384	3.72	9.68						
$LiBF_4$/PC=1/100	0.010	2.5	1.200	0.117	2.01	17.23						
(LiTfO/PC=1/2.5)**	0.286	355	1.407	3.422	0.12	0.04						0.827
LiTfO/PC=1/3	0.250	186	1.389	3.005	0.297	0.10	0.50	1.86	0.40	0.33	0.30	0.758
LiTfO/PC=1/4	0.200	52	1.351	2.394	0.541	0.23	1.41	4.78	1.31	1.01	0.22	0.675
LiTfO/PC=1/6	0.143	16	1.309	1.703	1.147	0.67	4.12	11.66	3.84	2.94	0.23	
LiTfO/PC=1/8	0.111	9.5	1.282	1.318	1.586	1.20	6.84	18.84	7.97	5.47	0.22	
LiTfO/PC=1/10	0.091	7.0	1.267	1.076	1.875	1.74	8.27	22.48	9.31	6.494	0.27	0.576
LiTfO/PC=1/15	0.063	4.8	1.244	0.737	2.147	2.91						
LiTfO/PC=1/30	0.032	3.3	1.220	0.379	2.084	5.50						
LiTfO/PC=1/50	0.020	2.9	1.210	0.230	1.893	8.23						
LiTfO/PC=1/90	0.011	2.6	1.203	0.129	1.42	11.06						
pure PC		2.3	1.195									

*For ClO_4-based electrolytes, diffusion measurements (PFG–NMR) were not feasible; therefore, D_{anion}, Λ_{NMR}, and $\Lambda_{imp}/\Lambda_{NMR}$ were not determined.

**PFG–NMR could not be performed because LiTfO/PC=1/2.5 exists as a supercooled liquid and readily solidifies.

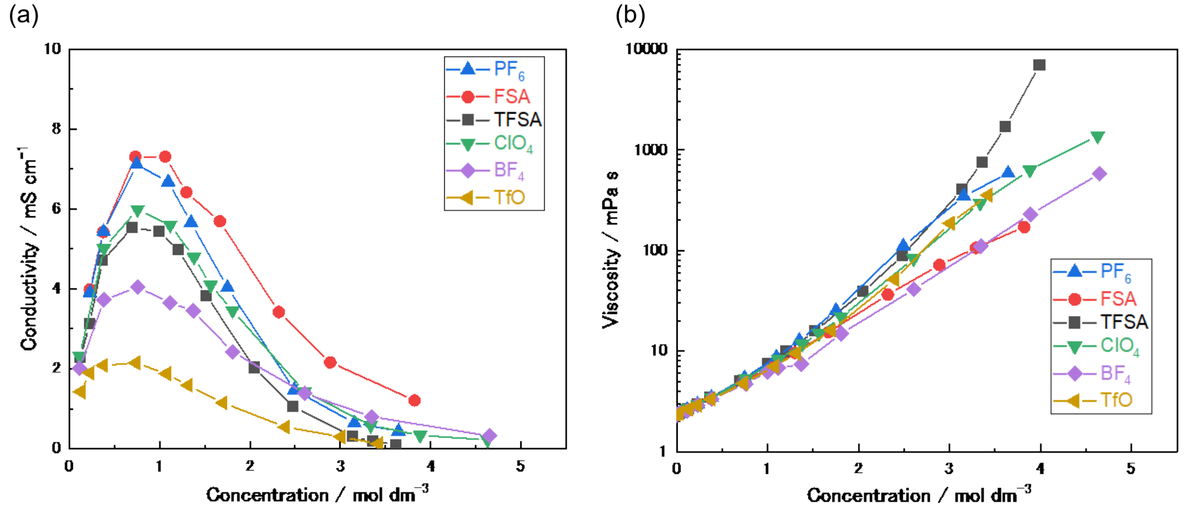

Figure S4 (a) Ionic conductivity and (b) viscosity as a function of lithium salt concentration for the LiX/PC solutions measured at 30 °C.

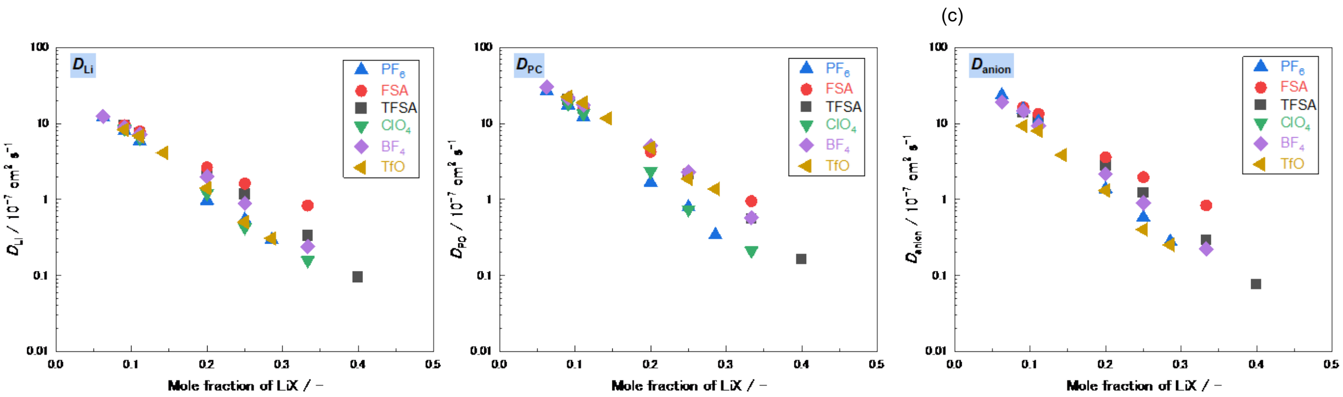

Figure S5 Self-diffusion coefficients of (a) Li^+ (D_{Li}), (b) PC (D_{PC}), and (c) the anion (D_{anion}) as a function of the lithium salt mole fraction in LiX/PC solutions at 30 °C.

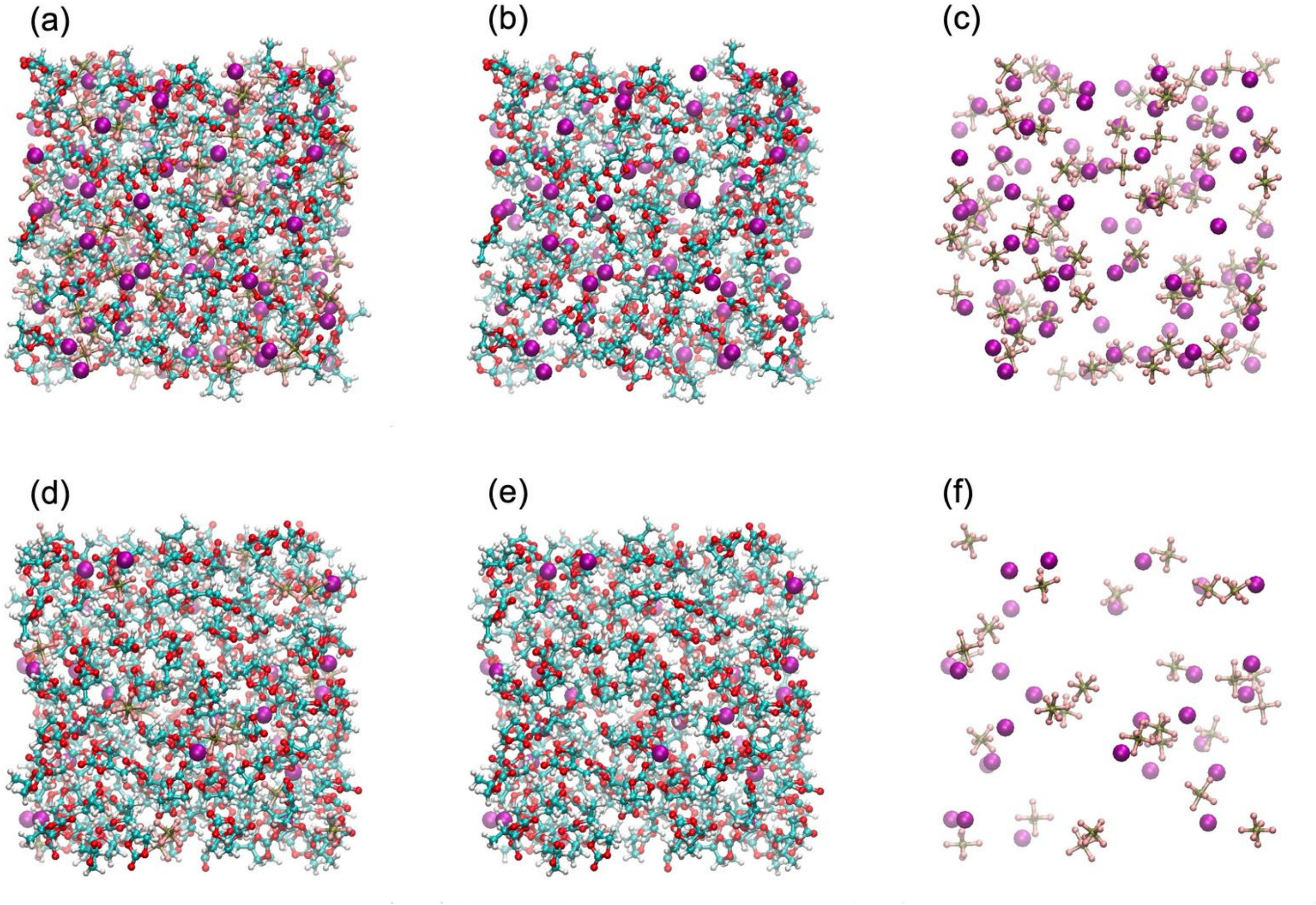

Figure S6 Snapshots of the $LiPF_6$ solution. Carbon, hydrogen, oxygen, fluorine, phosphorus, and lithium atoms are shown in light blue, white, red, pink, brown, and purple, respectively. Li^+ ions are represented using a space-filling model, whereas other atoms are shown using a ball-and-stick model: (a–c) concentrated solutions of the $LiPF_6$ solution ($LiPF_6$/PC = 1/2.5); (d–f) dilute $LiPF_6$ solution ($LiPF_6$/PC = 1/8); (a), (d) all molecules are shown; (b), (e) Li^+ ions and PC are shown; and (c), (f) Li^+ ions and anions are shown.

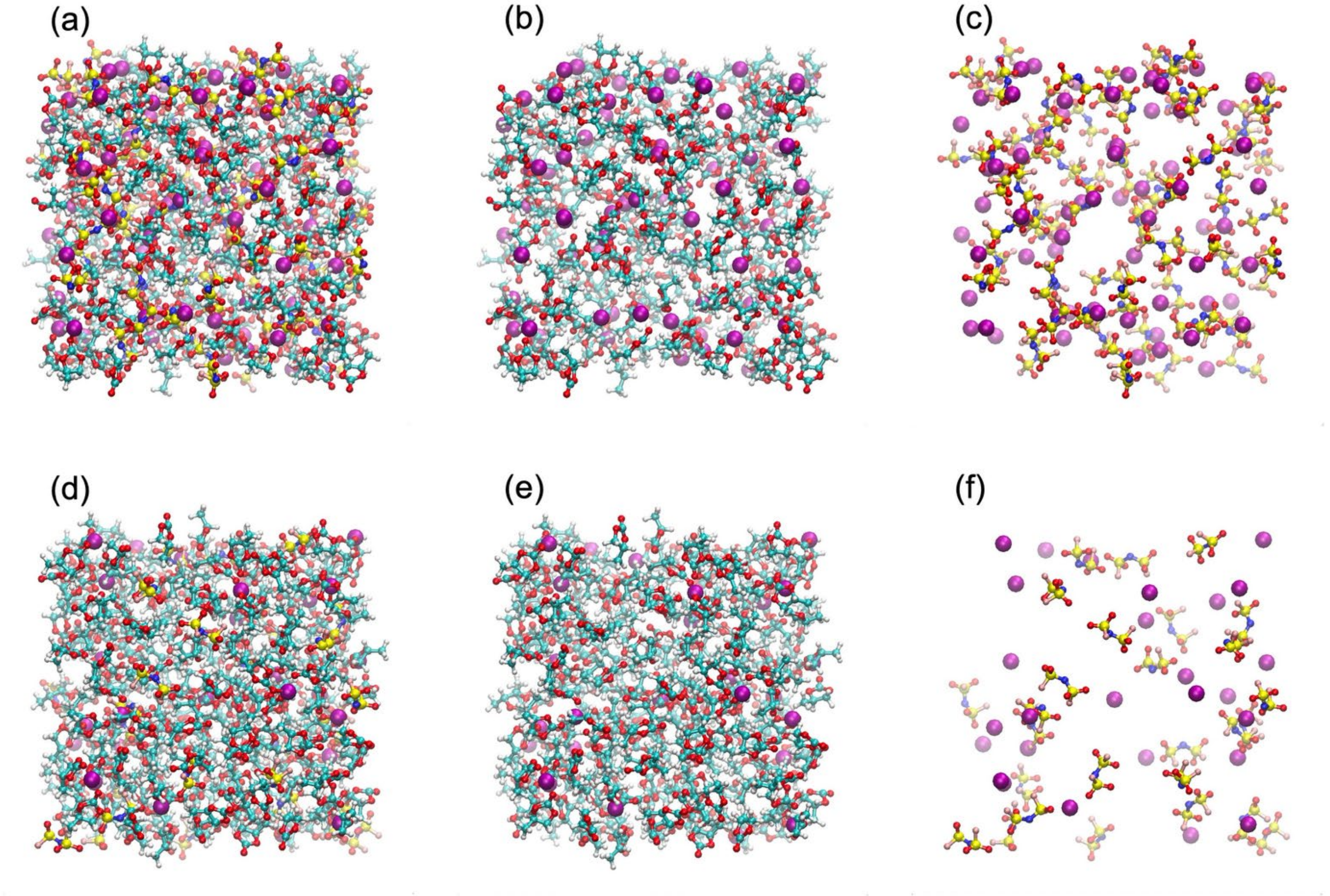

Figure S7 Snapshots of the LiFSA solution. Carbon, hydrogen, oxygen, nitrogen, fluorine, sulfur, and lithium atoms are shown in light blue, white, red, blue, pink, yellow, and purple, respectively. Li^+ ions are represented using a space-filling model, whereas other atoms are shown using a ball-and-stick model: (a–c) concentrated solutions of the LiFSA solution (LiFSA/PC = 1/2.5); (d–f) dilute LiFSA solution (LiFSA/PC = 1/8); (a), (d) all molecules are shown; (b), (e) Li^+ ions and PC are shown; and (c), (f) Li^+ ions and anions are shown.

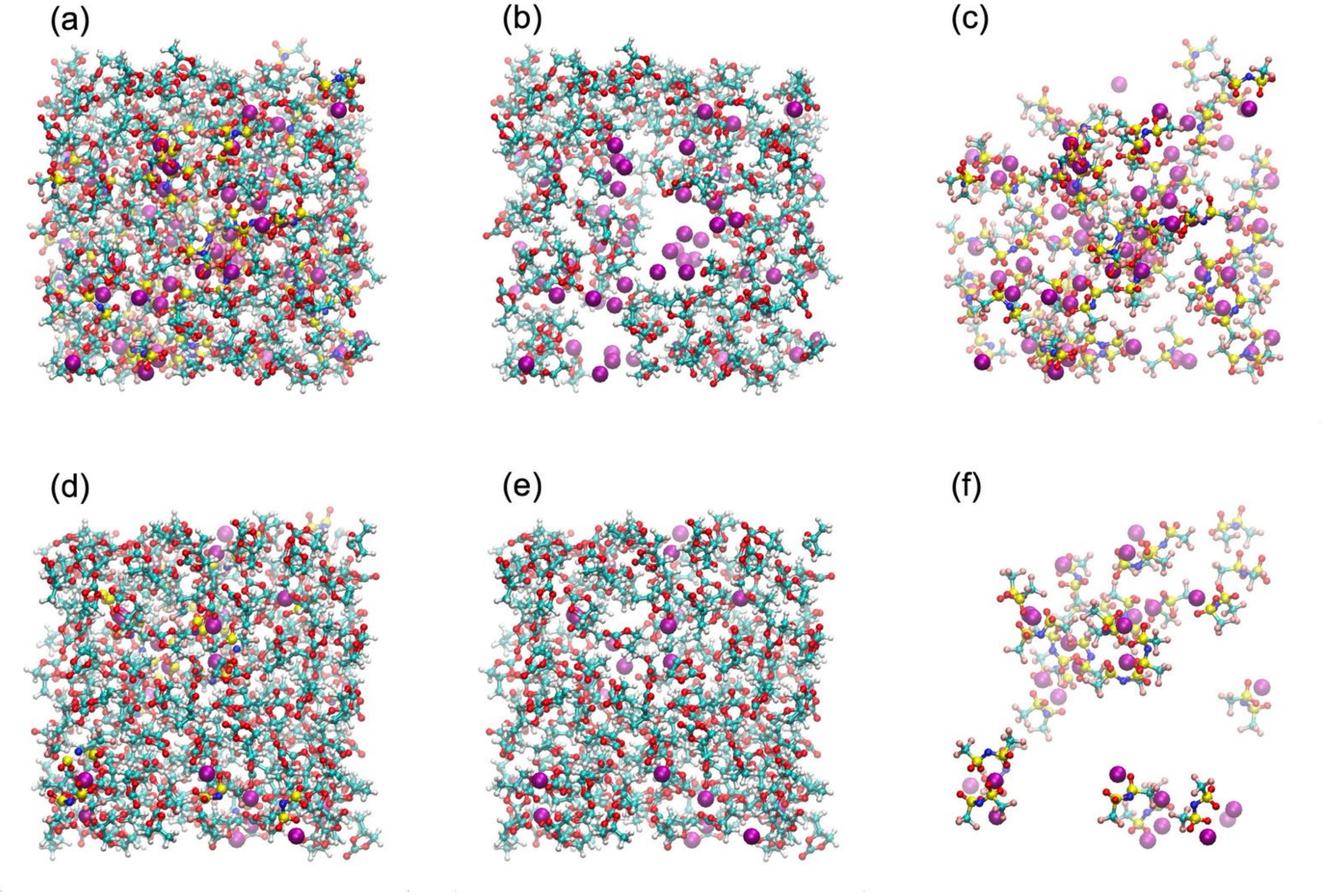

Figure S8 Snapshots of the LiTFSA solution. Carbon, hydrogen, oxygen, nitrogen, fluorine, sulfur, and lithium atoms are shown in light blue, white, red, blue, pink, yellow, and purple, respectively. Li^+ ions are represented using a space-filling model, whereas other atoms are shown using a ball-and-stick model: (a–c) concentrated solutions of the LiTFSA solution (LiTFSA/PC = 1/2.5); (d–f) dilute LiTFSA solution (LiTFSA/PC = 1/8); (a), (d) all molecules are shown; (b), (e) Li^+ ions and PC are shown; and (c), (f) Li^+ ions and anions are shown.

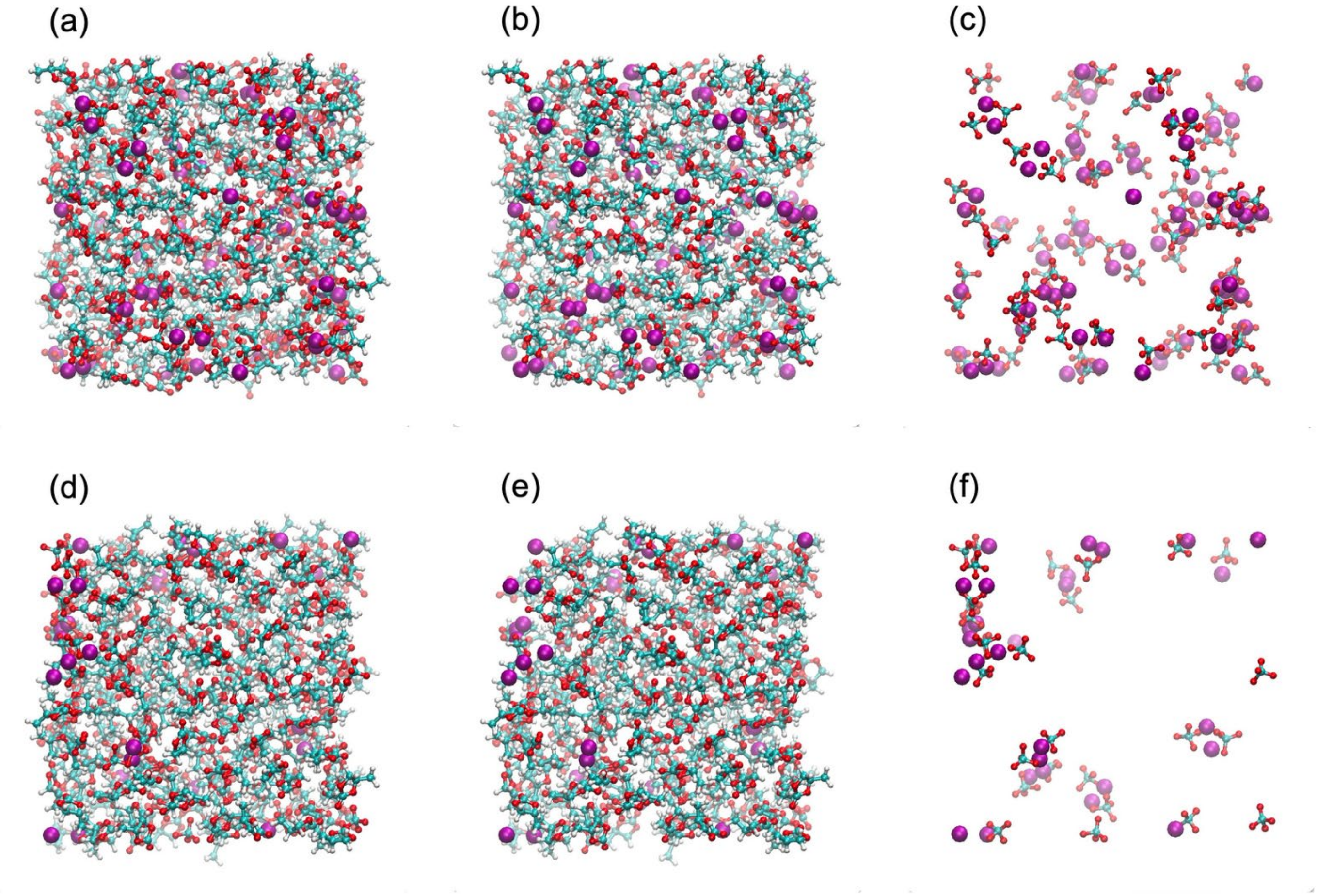

Figure S9 Snapshots of the $LiClO_4$ solution. Carbon, hydrogen, oxygen, chlorine, and lithium atoms are shown in light blue, white, red, blue, and purple, respectively. Li^+ ions are represented using a space-filling model, whereas other atoms are shown using a ball-and-stick model: (a–c) concentrated solutions of the $LiClO_4$ solution ($LiClO_4$/PC = 1/2.5); (d–f) dilute $LiClO_4$ solution ($LiClO_4$/PC = 1/8); (a), (d) all molecules are shown; (b), (e) Li^+ ions and PC are shown; and (c), (f) Li^+ ions and anions are shown.

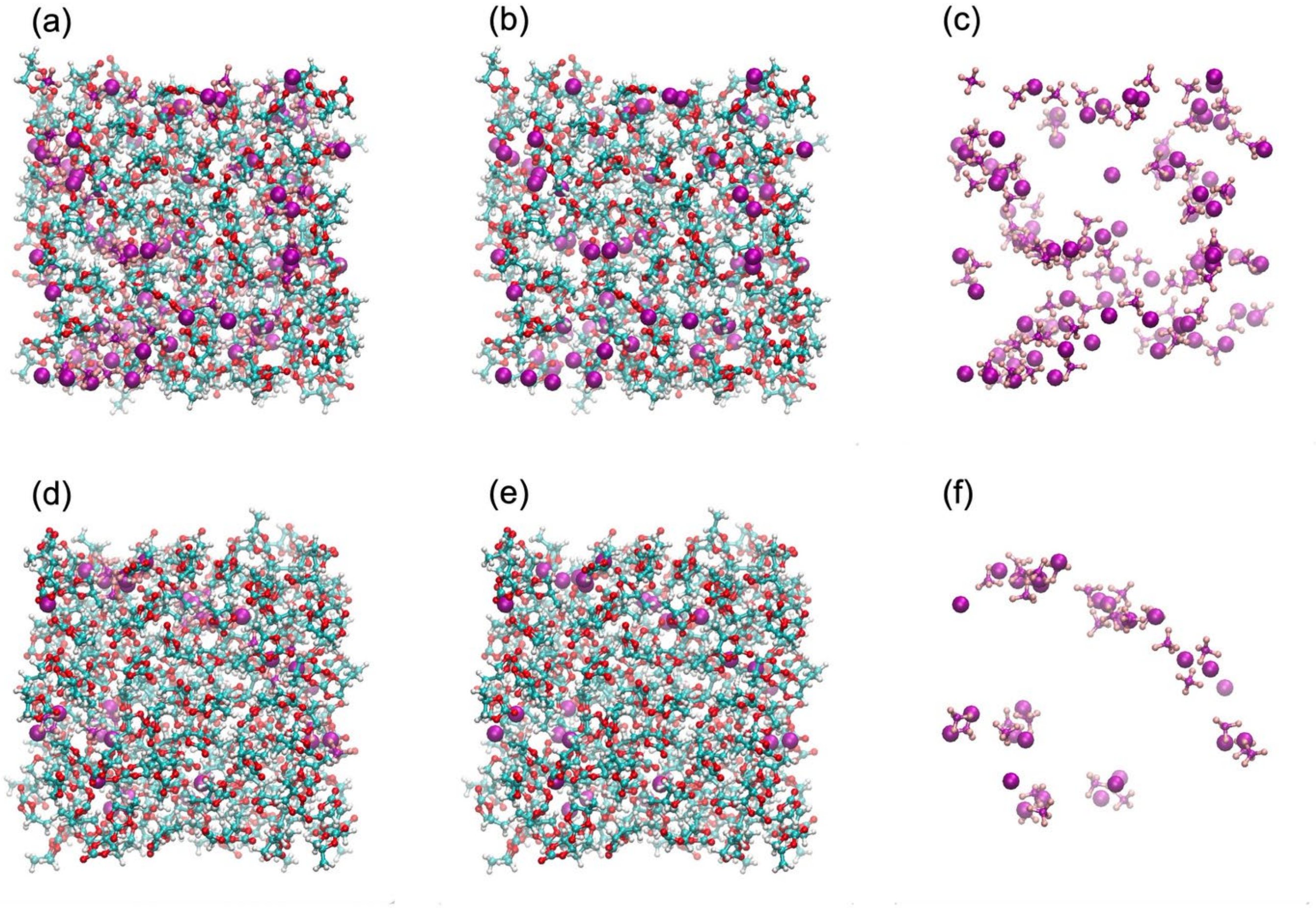

Figure S10 Snapshots of the $LiBF_4$ solution. Carbon, hydrogen, oxygen, fluorine, boron, and lithium atoms are shown in light blue, white, red, pink, purple, and purple, respectively. Li^+ ions are represented using a space-filling model, whereas other atoms are shown using a ball-and-stick model: (a–c) concentrated solutions of the $LiBF_4$ solution ($LiBF_4$/PC = 1/2.5); (d–f) dilute $LiBF_4$ solution ($LiBF_4$/PC = 1/8); (a), (d) all molecules are shown; (b), (e) Li^+ ions and PC are shown; and (c), (f) Li^+ ions and anions are shown.

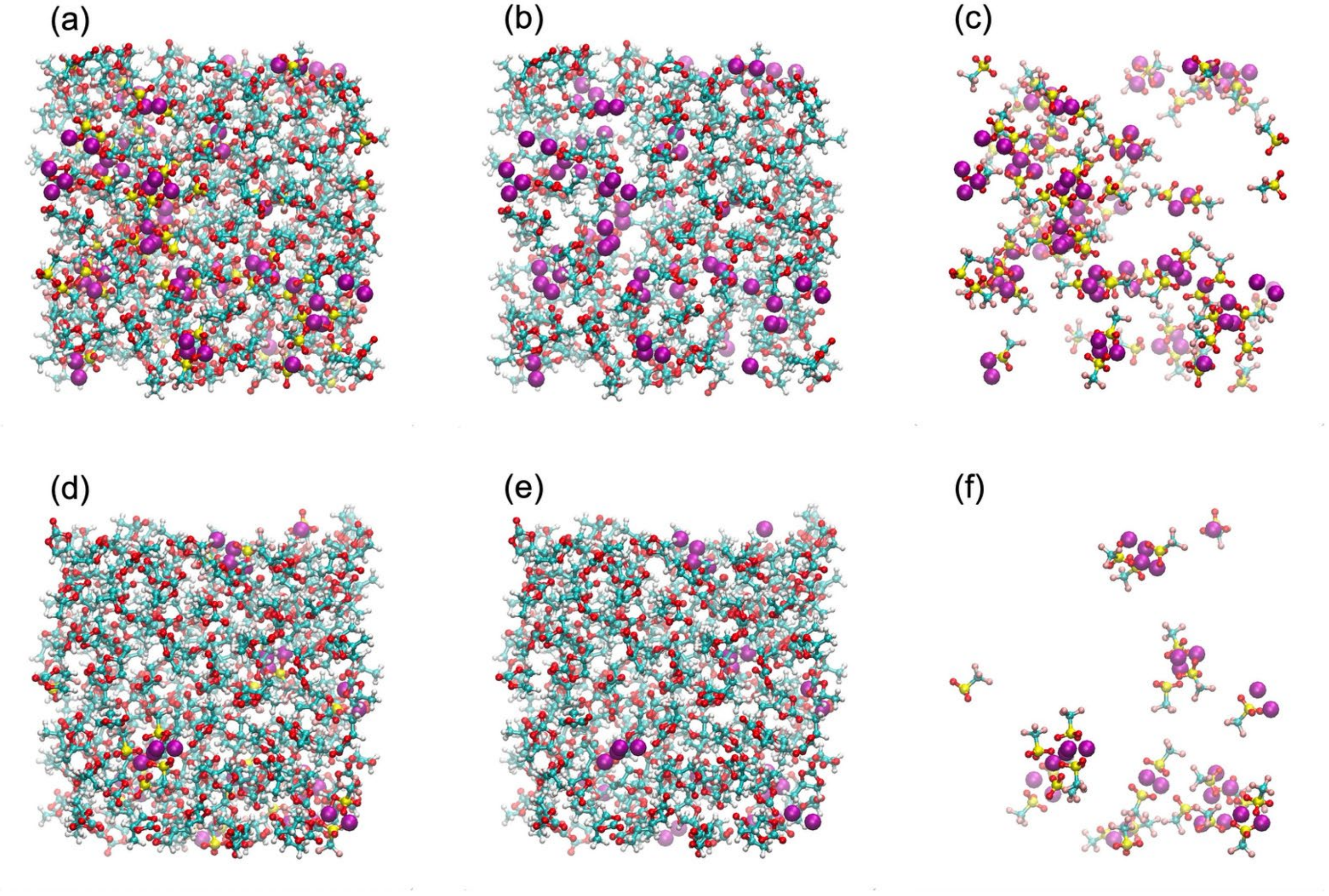

Figure S11 Snapshots of the LiTfO solution. Carbon, hydrogen, oxygen, nitrogen, fluorine, sulfur, and lithium atoms are shown in light blue, white, red, blue, pink, yellow, and purple, respectively. Li^+ ions are represented using a space-filling model, whereas other atoms are shown using a ball-and-stick model: (a–c) concentrated solutions of the LiTfO solution (LiTfO/PC = 1/2.5); (d–f) dilute LiTfO solution (LiTfO/PC = 1/8); (a), (d) all molecules are shown; (b), (e) Li^+ ions and PC are shown; and (c), (f) Li^+ ions and anions are shown.

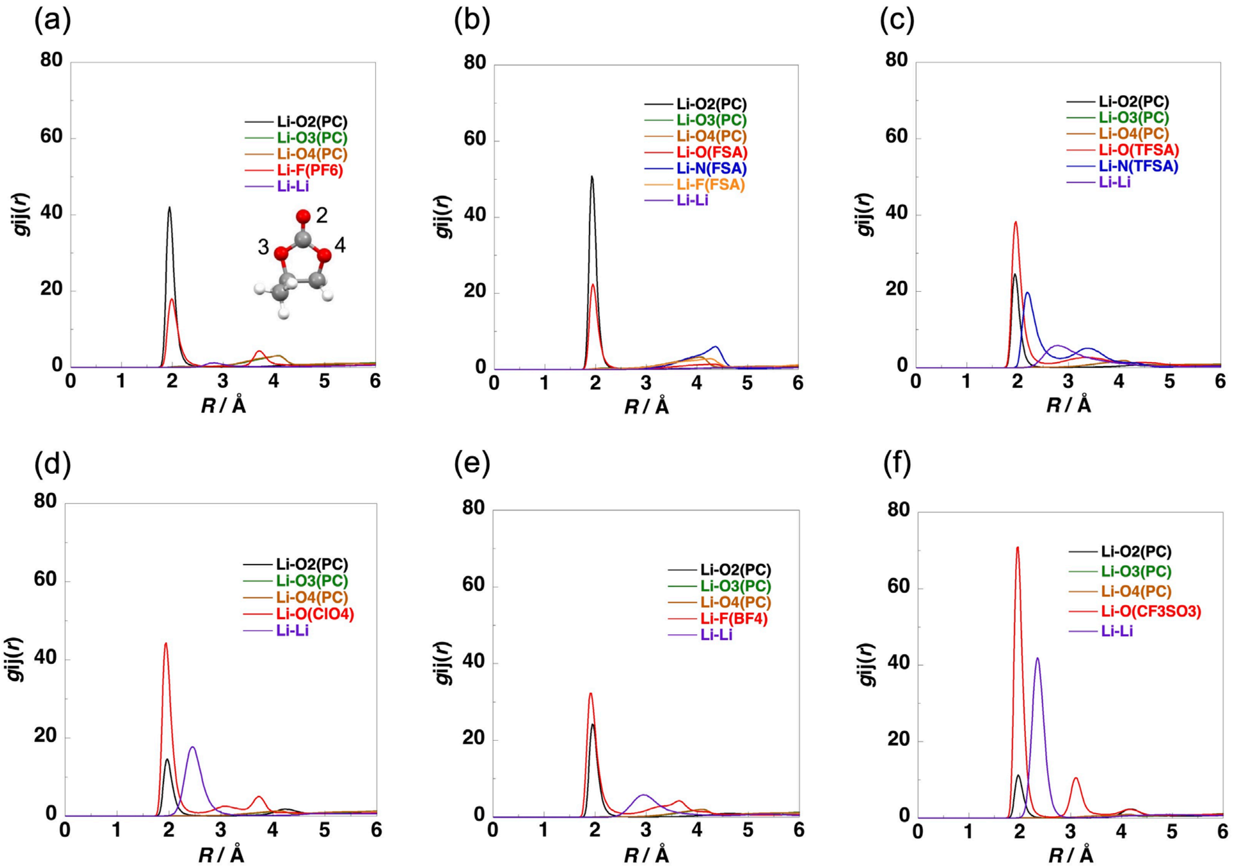

Figure S12 Site–site intermolecular radial distribution functions (RDFs) for the concentrated solutions of lithium salts (LiX). [LiX]/[PC] ratios are 1/2.5: (a) X = PF_6; (b) X = FSA; (c) X = TFSA, (d) X = ClO_4; (e) X = BF_4; and (f) X = TfO.

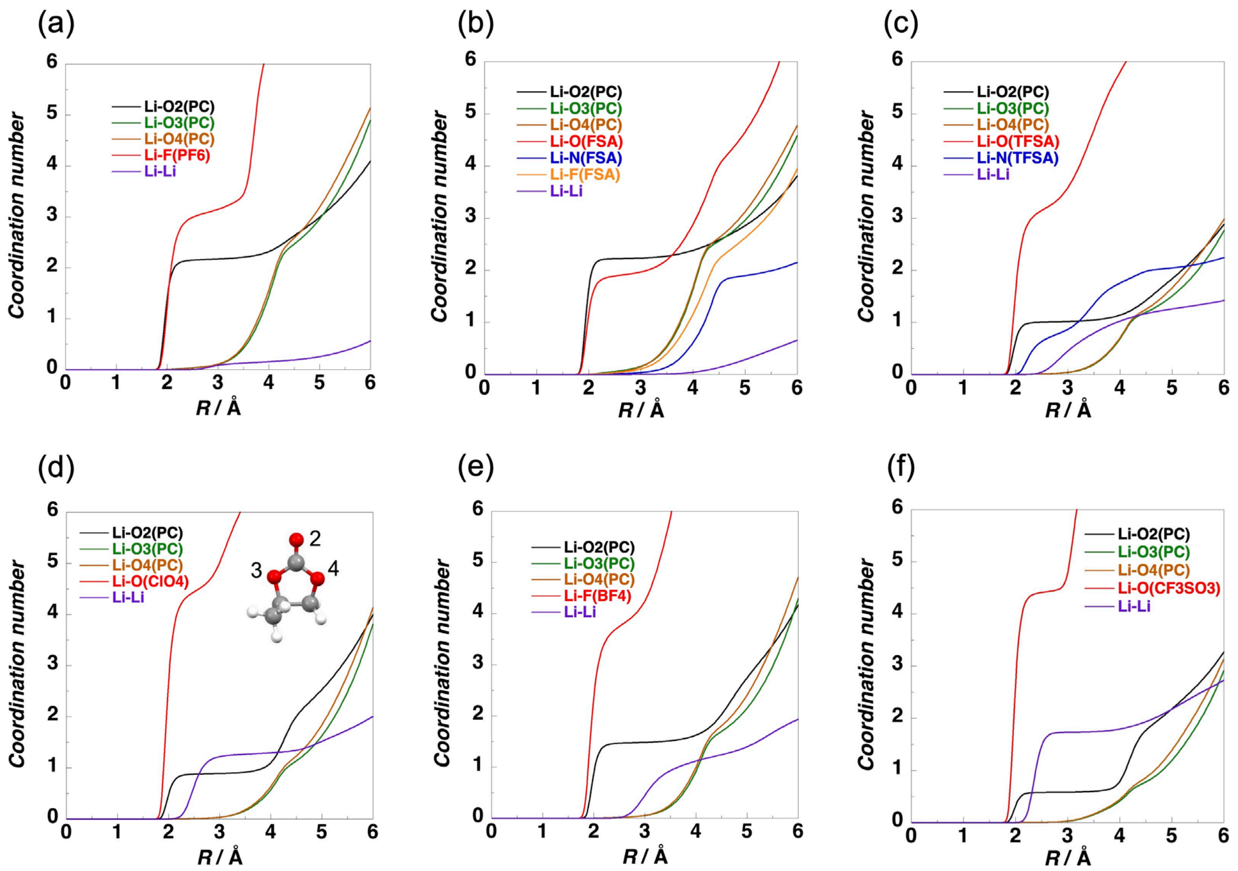

Figure S13 Cumulative coordination numbers around Li^+ ions for the concentrated solutions of LiX. [LiX]/[PC] ratios are 1/2.5: (a) X = PF_6; (b) X = FSA; (c) X = TFSA, (d) X = ClO_4; (e) X = BF_4; and (f) X = TfO.

Table S3 Calculated densities of the electrolytes and self-diffusion coefficients of the ions and PC in electrolytes.[a]

	ρ[b]	D[c]		
		Li	PC	Anion
$LiPF_6$/PC = 1/2.5	1.41	1.3	1.7	1.7
LiFSA/PC = 1/2.5	1.47	0.38	0.55	0.37
LiTFSA/PC = 1/2.5	1.49	1.1	3.3	1.1
$LiClO_4$/PC = 1/2.5	1.32	7.1	14	7.1
$LiBF_4$/PC = 1/2.5	1.35	1.3	2.8	1.5
LiOfT/PC = 1/2.5	1.37	0.38	0.55	0.37
$LiPF_6$/PC = 1/8	1.29	3.4	6.3	4.2
LiFSA/PC = 1/8	1.31	4.0	7.1	5.3
LiTFSA/PC = 1/8	1.33	3.6	11	4.2
$LiClO_4$/PC = 1/8	1.25	9.3	17	9.4
$LiBF_4$/PC = 1/8	1.25	4.0	13	4.1
LiOfT/PC = 1/8	1.27	6.1	17	6.8

[a] Calculated at 303.15 K

[b] Density in g cm^{-3}.

[c] Self-diffusion coefficient in 10^{-7} cm^2 s^{-1}.

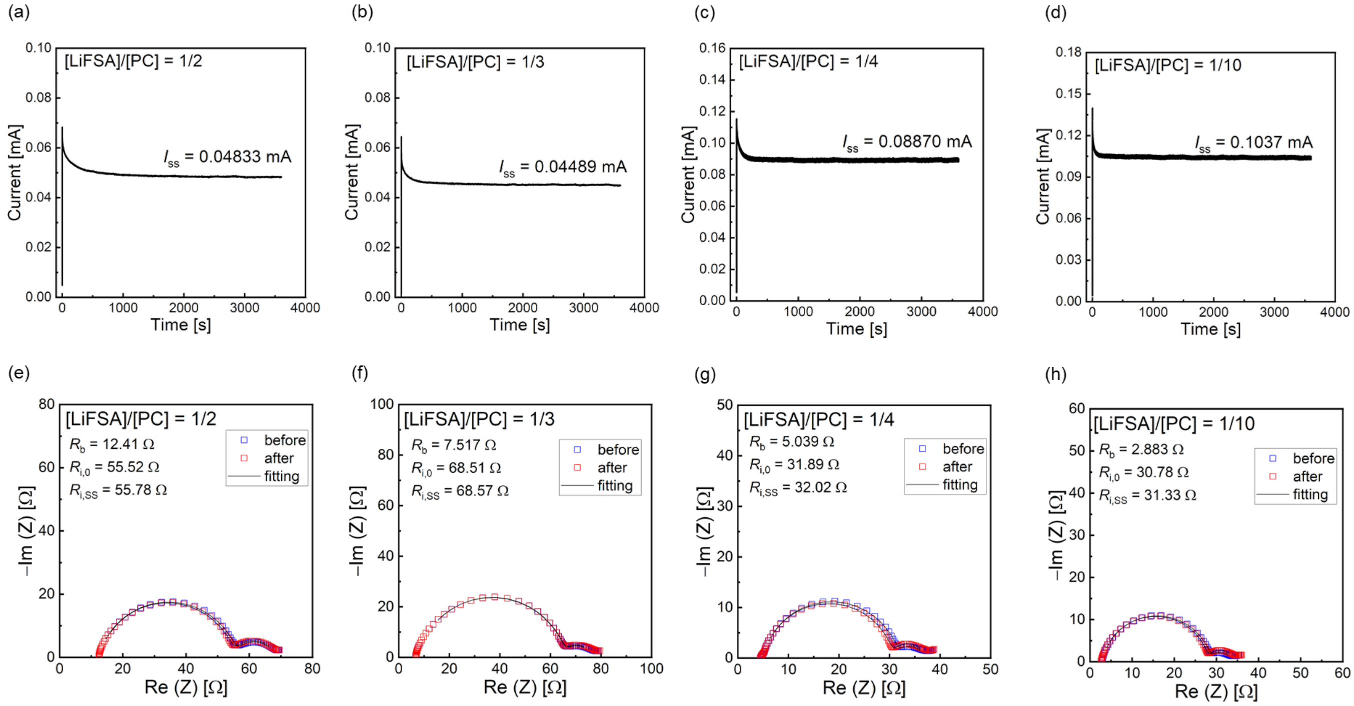

Figure S14 Representative (a–d) chronoamperograms and (e–h) Nyquist plots of Li symmetric cells measured at 30 ℃ before and after polarization using electrolytes with **LiFSA**/PC molar ratios of (a, e) 1/2, (b, f) 1/3, (c, g) 1/4, and (d, h) 1/10. The data shown in **Figure 3** represent the average of three independent measurements. For Nyquist plots exhibiting two semicircles, the combined resistance of both semicircles is defined as R_i.

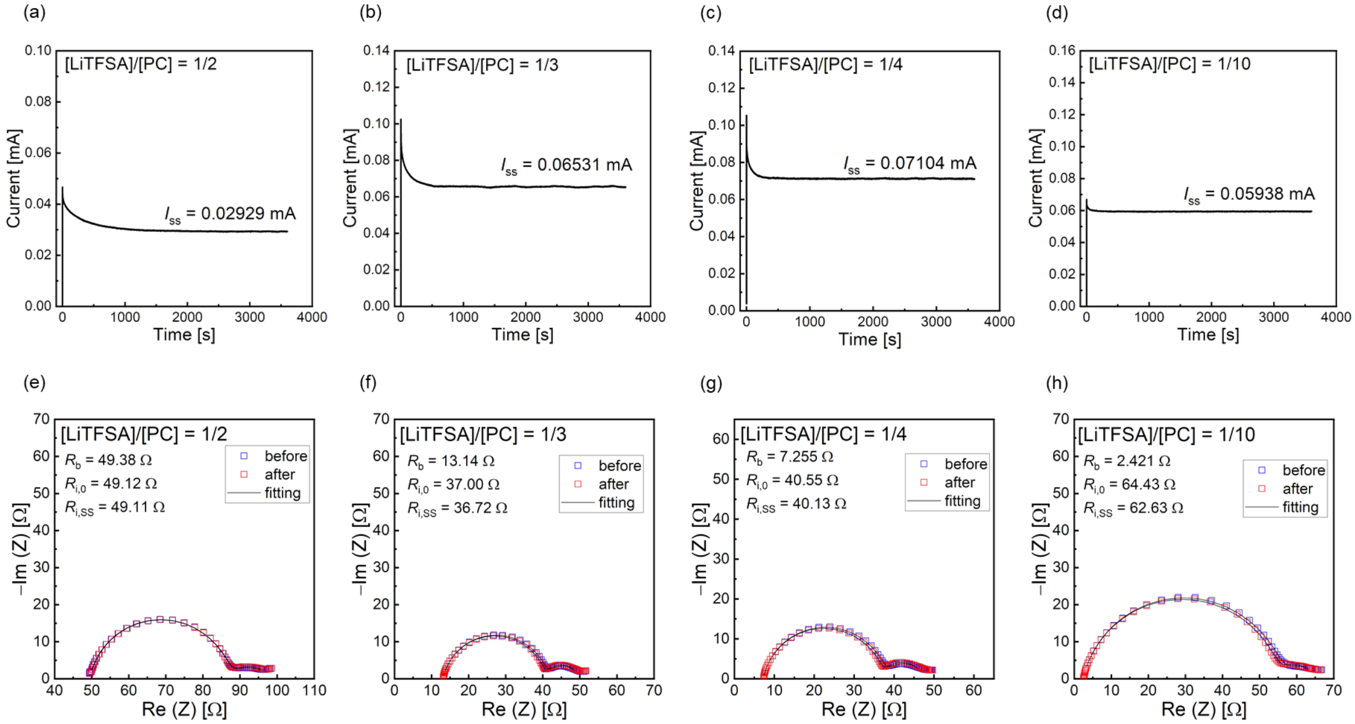

Figure S15 Representative (a–d) chronoamperograms and (e–h) Nyquist plots of Li symmetric cells measured at 30 ℃ before and after polarization using electrolytes with **LiTFSA**/PC molar ratios of (a, e) 1/2, (b, f) 1/3, (c, g) 1/4, and (d, h) 1/10. The data shown in **Figure 3** represent the average of three independent measurements. For Nyquist plots exhibiting two semicircles, the combined resistance of both semicircles is defined as R_i.

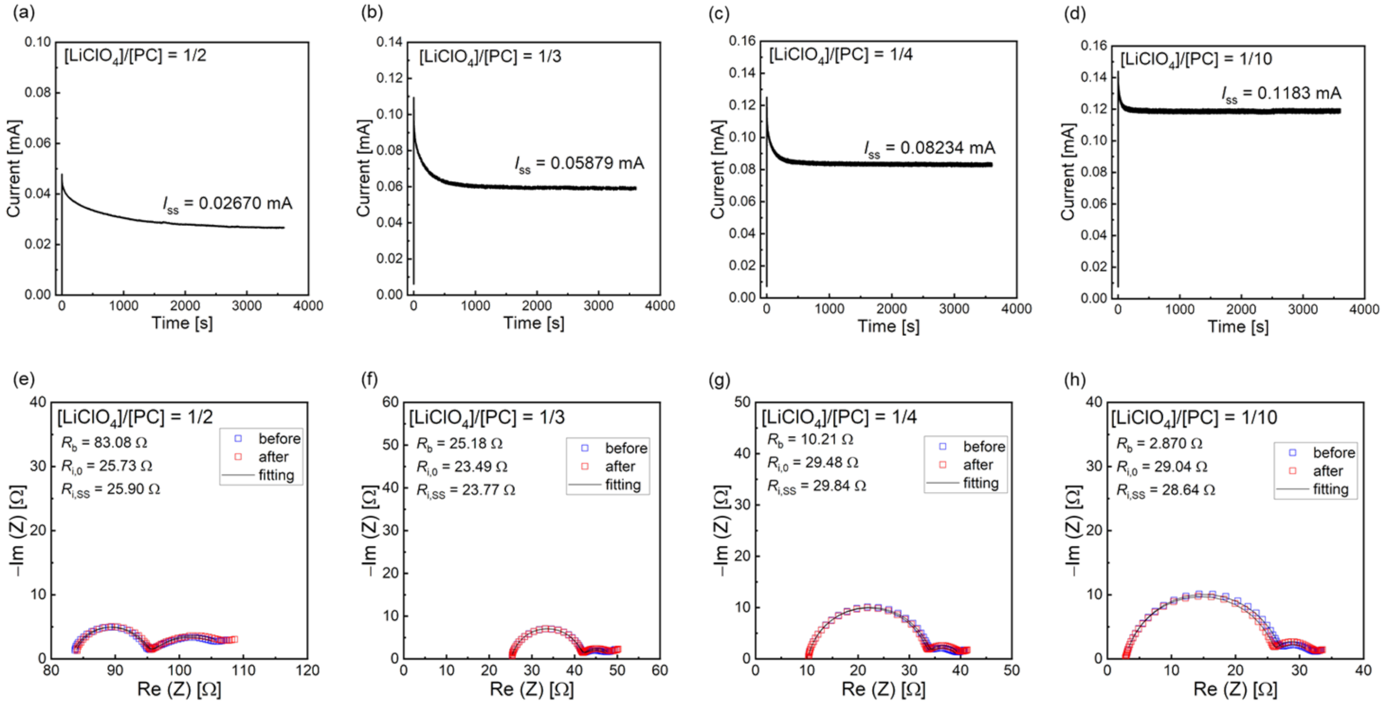

Figure 16 Representative (a–d) chronoamperograms and (e–h) Nyquist plots of Li symmetric cells measured at 30 ℃ before and after polarization using electrolytes with **$LiClO_4$**/PC molar ratios of (a, e) 1/2, (b, f) 1/3, (c, g) 1/4, and (d, h) 1/10. The data shown in **Figure 3** represent the average of three independent measurements. For Nyquist plots exhibiting two semicircles, the combined resistance of both semicircles is defined as R_i.

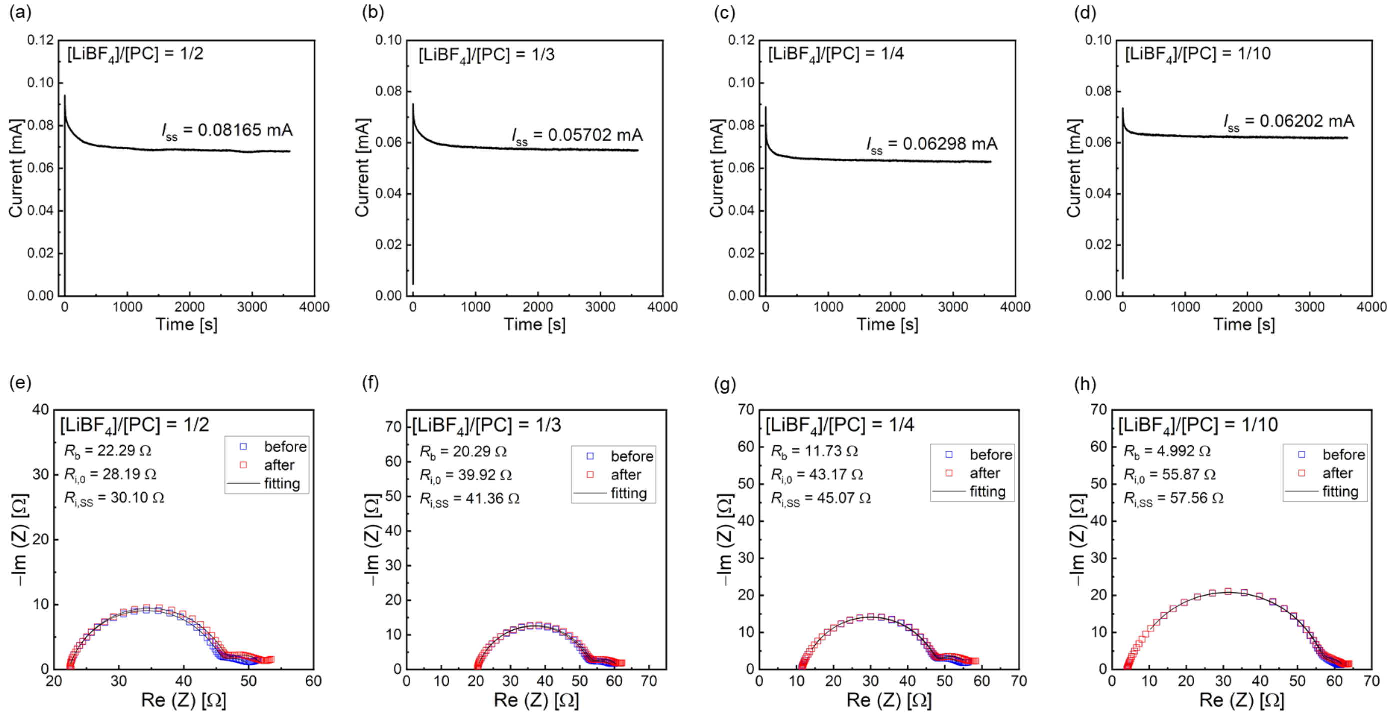

Figure S17 Representative (a–d) chronoamperograms and (e–h) Nyquist plots of Li symmetric cells measured at 30 ℃ before and after polarization using electrolytes with **$LiBF_4$**/PC molar ratios of (a, e) 1/2, (b, f) 1/3, (c, g) 1/4, and (d, h) 1/10. The data shown in **Figure 3** represent the average of three independent measurements. For Nyquist plots exhibiting two semicircles, the combined resistance of both semicircles is defined as R_i.

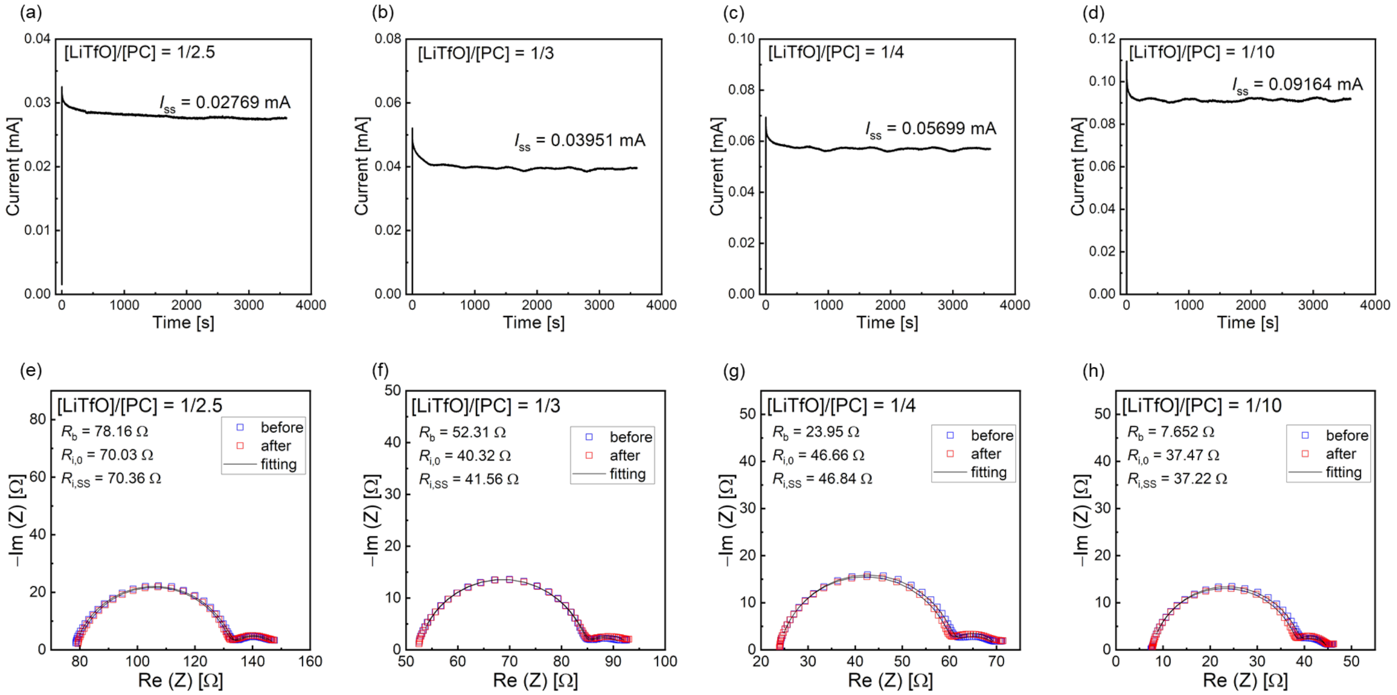

Figure S18 Representative (a–d) chronoamperograms and (e–h) Nyquist plots of Li symmetric cells measured at 30 ℃ before and after polarization using electrolytes with **LiTfO**/PC molar ratios of (a, e) 1/2, (b, f) 1/3, (c, g) 1/4, and (d, h) 1/10. The data shown in **Figure 3** represent the average of three independent measurements. For Nyquist plots exhibiting two semicircles, the combined resistance of both semicircles is defined as R_i.
